# Dynamic Regulation of Hydrogen Bonding Networks and Solvation Structures for Synergistic Solar-Thermal Desalination of Seawater and Catalytic Degradation of Organic Pollutants

**DOI:** 10.1007/s40820-024-01544-9

**Published:** 2024-10-23

**Authors:** Ming-Yuan Yu, Jing Wu, Guang Yin, Fan-Zhen Jiao, Zhong-Zhen Yu, Jin Qu

**Affiliations:** 1 Beijing Key Laboratory of Advanced Functional Polymer Composites, Beijing University of Chemical Technology, Beijing, 100029 People’s Republic of China; 2 State Key Laboratory of Organic-Inorganic Composites, College of Materials Science and Engineering, Beijing University of Chemical Technology, Beijing, 100029 People’s Republic of China

**Keywords:** Solar steam generation, Seawater desalination, Catalytic degradation, Bacterial cellulose, Cobalt hydroxycarbonate nanorods

## Abstract

Although solar steam generation strategy is efficient in desalinating seawater, it is still challenging to achieve continuous solar-thermal desalination of seawater and catalytic degradation of organic pollutants. Herein, dynamic regulations of hydrogen bonding networks and solvation structures are realized by designing an asymmetric bilayer membrane consisting of a bacterial cellulose/carbon nanotube/Co_2_(OH)_2_CO_3_ nanorod top layer and a bacterial cellulose/Co_2_(OH)_2_CO_3_ nanorod (BCH) bottom layer. Crucially, the hydrogen bonding networks inside the membrane can be tuned by the rich surface –OH groups of the bacterial cellulose and Co_2_(OH)_2_CO_3_ as well as the ions and radicals in situ generated during the catalysis process. Moreover, both SO_4_^2−^ and HSO_5_^−^ can regulate the solvation structure of Na^+^ and be adsorbed more preferentially on the evaporation surface than Cl^−^, thus hindering the de-solvation of the solvated Na^+^ and subsequent nucleation/growth of NaCl. Furthermore, the heat generated by the solar-thermal energy conversion can accelerate the reaction kinetics and enhance the catalytic degradation efficiency. This work provides a flow-bed water purification system with an asymmetric solar-thermal and catalytic membrane for synergistic solar thermal desalination of seawater/brine and catalytic degradation of organic pollutants.

**Supplementary Information:**

The online version contains supplementary material available at 10.1007/s40820-024-01544-9.

## Highlights

- A flow-bed water purification system with an asymmetric solar-thermal and catalytic membrane is designed for synergistic solar-thermal desalination of seawater/brine and catalytic degradation of organic pollutants.
- The hydrogen bonding networks can be regulated by the abundant surface –OH groups and the in situ generated ions and radicals during the degradation process for promoting solar-driven steam generation.
- The de-solvation of solvated Na^+^ and subsequent nucleation/growth of NaCl are effectively inhibited by SO_4_^2−^/HSO_5_^−^ ions.

**Supplementary Information:**

The online version contains supplementary material available at 10.1007/s40820-024-01544-9.

## Graphical abstract

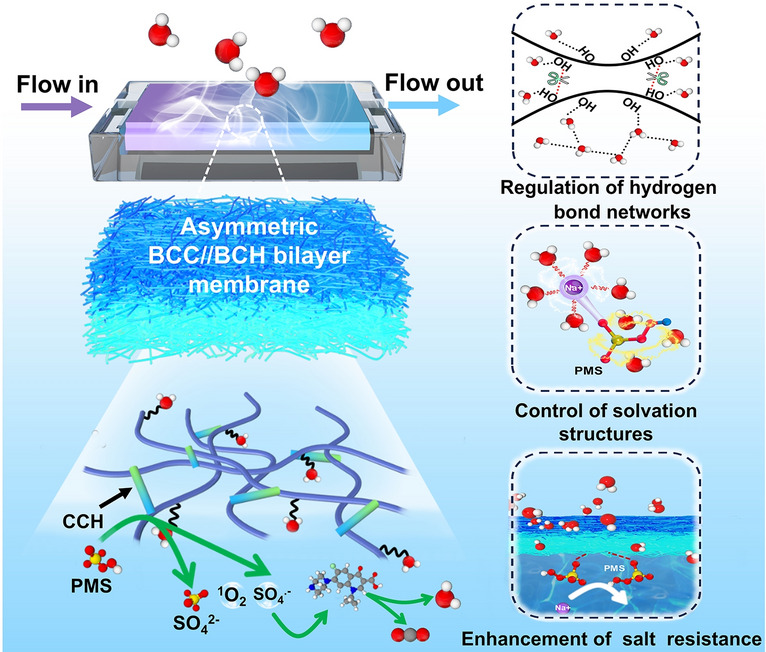

## Introduction

Energy crisis, environmental pollution, and the shortage of clean water have adversely affected the survival of mankind and the development of human society [1, 2]. The utilization of clean and renewable energy has become a global consensus for alleviating the above issues [3–8]. In particular, solar energy has distinct advantages in terms of abundance and sustainability [9–12]. Solar-driven desalination of seawater is environmentally friendly and provides an effective solution for generating clean water [13–21]. Although most solar-driven interface evaporators can realize seawater desalination and remove some of organic pollutants during the solar steam generation process, the catalytic degradation of organic contaminants in seawater, including antibiotics, dyes, and volatile organic pollutants, has not been well solved [21, 22,23]. The discharge of antibiotics and other organic pollutants into the seawater has caused serious environmental problems. The total absolute abundance of antibiotic resistance genes in the Bohai coastal water is about 1–4 orders of magnitude higher than in the ocean [24]. In addition, the temperature of the air–water interface can reach 40‒70 °C under 1-sun irradiation, which may cause water quality problems because the volatile organic compounds (VOCs) are more favorably enriched in the condensed water [25]. It is therefore necessary to integrate efficient catalytic degradation with solar-driven desalination for obtaining clean water from seawater containing both salt ions and organic contaminants.

Basically, the hydrogen bonding network is crucial for solar-thermal desalination, and the hydration and solvation structures of various ions and molecules strongly influence their adsorption and desorption on the catalyst surfaces [26–28, 28]. To achieve superb solar-thermal desalination of seawater/brine and catalytic degradation of organic pollutants therein, it is imperative to elucidate the regulatory rules of hydrogen bonding networks in a solar-driven interface evaporator and the solvation structures of various inorganic ions and organic pollutant molecules.

As reported, advanced oxidation processes (AOPs) are effective in degrading various organic pollutants by activating peroxomonosulfate (PMS) to generate highly active SO_4_^•−^, ^•^OH, singlet oxygen (^1^O_2_), and other reactive oxygen species (ROS), ultimately achieving efficient catalytic degradation of the pollutants [29]. It is expected that if water molecules are adsorbed or transferred to the catalyst surface, the hydrogen bonding between the water molecules would be affected and hence the water vaporization enthalpy would vary accordingly. Generally, the hydrogen bonding of the exposed active sites of the catalyst with water is much stronger than that between water molecules, which would break the hydrogen bonding of the water itself, leading to a lower vaporization enthalpy of the water [30]. Additionally, AOPs can introduce some inorganic ions or radicals into the water, which are beneficial for the catalytic degradation of organic pollutants as well as for the regulation of surface charges and hydration shells of solvated ions, thereby varying the nearby hydrogen bonds with water. Besides, these species can be adsorbed on the evaporation surface, further affecting the hydrogen bonding at the catalyst surface. Therefore, the regulation of interfacial hydrogen bonding and solvation structures can highly adjust the water states, which is closely related to the solar-driven water evaporation efficiency of the solar steam generation system.

Apparently, salt crystallization on the evaporation surface would adversely affect the efficiency and continuity of the solar-driven desalination of seawater/brine [31–33], because the solid salts can overshadow the solar-thermal conversion surface, block the internal water supply channels, and prevent vapor diffusion and escape [34–37]. Generally, a salt crystallization process involves the de-solvation of the solvated Na^+^ at the evaporation surface, followed by the nucleation and growth of salts. Both the desalination and the AOP catalysis involve the adsorption of substances or ions onto the surfaces of solar-thermal conversion materials/catalysts. Therefore, these inorganic ions or ROS dynamically generated during the AOPs catalytic degradation may enter the hydration shell of the solvated Na^+^ to slow down the de-solvation and be adsorbed on the surface of the solar-thermal conversion material/catalyst to hinder subsequent crystallization, thus promoting desalination performances.

**Fig. 1:**
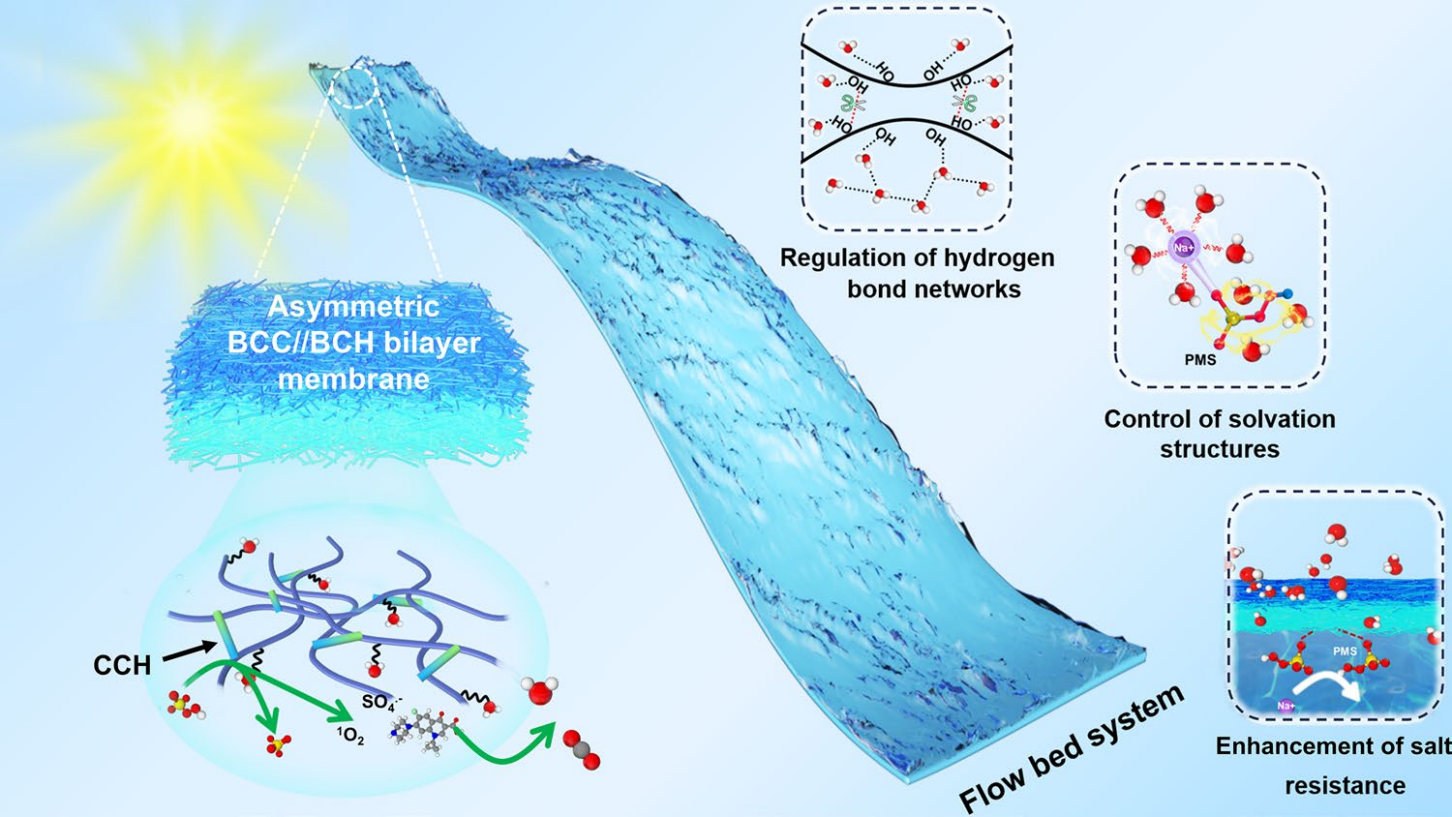
Design of a bilayer membrane-based flow-bed water purification system for continuous and simultaneous solar-thermal desalination of brine and catalytic degradation of antibiotic contaminants

Herein, a bilayer membrane-based flow-bed water purification system is designed to realize the dynamic regulation of hydrogen bonding networks and solvation structures for continuous solar-thermal desalination of seawater/brine and simultaneous catalytic degradation of organic pollutants. The fabricated bilayer membrane consists of the bacterial cellulose/carbon nanotube/Co_2_(OH)_2_CO_3_ nanorod (BCC) top layer for solar-thermal energy conversion, water evaporation, and catalytic degradation of ciprofloxacin hydrochloride (CIP) pollutant, and the bacterial cellulose/Co_2_(OH)_2_CO_3_ nanorod (BCH) bottom layer that is immersed in water for fast water transfer and catalytic degradation of the CIP pollutant (Fig. 1). The in situ synthesized cobalt hydroxycarbonate (CCH) nanorods can catalytically degrade various organic pollutants via an AOPs degradation process, while the decorated carbon nanotubes (CNTs) serve as efficient solar-thermal energy conversion materials for solar-driven evaporation and desalination of seawater/brine. More importantly, the hydrogen bonding networks inside the membrane can be regulated by the abundant surface –OH groups of bacterial cellulose (BC) and CCHs as well as the ions and radicals in situ generated during the flow-bed AOPs catalytic degradation to increase the proportion of weakly bound water, thereby reducing the water vaporization enthalpy for promoting the solar steam generation efficiency. Furthermore, SO_4_^2−^ and HSO_5_^−^ anions can participate in the solvation structure of Na^+^ and be more preferentially adsorbed on the evaporation surface than Cl^−^, preventing the de-solvation of solvated Na^+^ and the nucleation/growth of NaCl, thus enhancing the salt-resistant efficiency during the solar-thermal desalination of seawater/brine.

To address the issues of low mass and heat transfer efficiencies, less sufficient solar light absorption of catalysts and pollutants, discontinuity in desalination, and low water production efficiency of the solar steam generation and catalysis process, a bilayer membrane-based flow-bed water purification system is designed to achieve synchronous and continuous solar-thermal desalination of seawater/brine and catalytic degradation of organic pollutants. As a result, the designed bilayer BCC//BCH membrane-based flow-bed purification system achieves a high water evaporation rate of 1.81 kg m^−2^ h^−1^ and a satisfactory CIP degradation efficiency of 92% under 1-sun solar light irradiation during purifying a wastewater with 50 ppm of CIP at a flow rate of 10 mL h^−1^. Even when purifying a flowing complex wastewater with 25 wt% of NaCl and 50 ppm of CIP, the flow-bed purification system still achieves a stable water evaporation rate (~ 1.88 kg m^−2^ h^−1^) and a CIP degradation efficiency (~ 94%). The relationships and synergies between the solar-thermal water evaporation and the catalytic degradation are explored in detail by combining first-principle calculations and simulations.

## Experimental Section

### Materials

Urea (Aladdin, CH_4_N_2_O), methylene blue (Aladdin, MB), methyl orange (Aladdin, MO), Rhodamine B (Fuchen, RhB), ciprofloxacin hydrochloride (Picasso, CIP), cobalt chloride hexahydrate (Alfa Aesar, CoCl_2_·6H_2_O), ethylene glycol (FuYu, EG), tert-butanol (Fuchen, TBA), ethanol (Innochem, EtOH), methanol (Innochem, MeOH), p-benzoquinone (Macklin, p-BQ), potassium peroxymonosulfate (Aladdin, 2KHSO_5_·3KHSO_4_·K_2_SO_4_, PMS), L-histidine (Macklin), aqueous bacterial cellulose suspension (Qihong Tech, BC, 7.5 wt%), aqueous dispersion of carbon nanotubes (XFNANO, CNTs, 0.15 wt%), 5,5-dimethyl-1-pyrroline N-oxide (DMPO), and 2,2,6,6-tetramethylpiperidine (TEMP) were all used as received.

### Preparation of Bilayer Membranes with a BCC Top Layer and a BCH Bottom Layer

The decoration of cobalt hydroxycarbonate nanorods (CCH) and CNTs on bacterial cellulose (BC) was carried out by synthesizing CCH nanorods with an ethylene glycol-assisted hydrothermal process in the presence of the CNTs and BC components. 2 mmol of CoCl_2_·6H_2_O and 5 mmol of CO(NH_2_)_2_ were dissolved in a mixture containing 12 mL of aqueous BC dispersion (2 mg mL^−1^), 18 mL of aqueous CNT dispersion (0.15 wt%), and 30 mL of ethylene glycol. The resulting suspension was transferred to a 100 mL autoclave for hydrothermal reaction at 140 °C for 4 h and centrifuged and washed three times with water and ethanol. The centrifuged precipitate was dispersed in water to form an aqueous suspension of BCC at a concentration of 3 mg mL^−1^. Similarly, the bacterial cellulose/cobalt hydroxycarbonate (BCH) suspension with a concentration of 5 mg mL^−1^ was prepared under the same conditions except that the aqueous CNT dispersion (0.15 wt%) was replaced by an equal volume of pure water. The BCC (4 mL) and BCH (6 mL) suspensions were vacuum filtered sequentially through a poly(tetrafluoroethylene) (PTFE) membrane filter (0.22 mm pore size, 47 mm in diameter), and the resulting bilayer membrane was dried at the ambient temperature, and peeled off from the PTFE filter. For comparison, a BC and CNT (BCT) membrane was prepared by vacuum filtration of the mixture containing 12 mL of aqueous BC dispersion (2 mg mL^−1^) and 18 mL of aqueous CNT dispersion (0.15 wt%). BCH and BCC membranes were prepared by vacuum filtration of 8 mL BCH dispersion (5 mg mL^−1^) and 14 mL BCC dispersion (3 mg mL^−1^), respectively.

### Characterization

Microstructures and morphologies were observed with a JEOL JEM-F200 transmission electron microscope (TEM) and a ZEISS Gemini SEM 300 scanning electron microscope (SEM) equipped with an energy-dispersive spectroscopy (EDS) system. The compositions of samples were characterized by a Thermo Scientific K-Alpha high-resolution X-ray photoelectron spectrometer (XPS), a Thermo Scientific Nicolet iS20 Fourier-transform infrared (FT-IR) spectrometer, and a Rigaku Smartlab SE X-ray diffractometer (XRD). Hydrophilicity was evaluated with a Krüss DSA30 contact angle system using 1 μL droplets as the indicator. Thermal conductivities of the samples were determined using a Hot Disk TPS 2500S apparatus. Absorption spectra of the samples were collected using a Shimadzu UV-3600 UV–Vis–NIR spectrophotometer. An electron paramagnetic resonance (EPR) spectrometer (Bruker A300-10/12) in continuous wave X-band mode was used to identify the dominant reactive species. Concentrations of the samples were measured with a Shimadzu UV2600 UV–Vis spectrophotometer and a Shimadzu LC 20A high-performance liquid chromatograph (HPLC) equipped with a C18 reversed-phase column.

### Solar Steam Generation Experiments

Sunlight was simulated using an optical assembly (AM 1.5G) of a CEL-HXUV300 Xenon lamp for solar-driven water evaporation. The solar light intensity can be adjusted by varying the current in the range of 14–16 A at 14 V. The evaporated water was weighed by an OHAUS CP214 electronic balance that was connected to a computer via an RS232 serial port. Surface temperatures of the samples were measured with an infrared camera (DMI220).

### Catalytic Degradation of BCC Powder under Stirring

Stirred catalytic oxidation was carried out as follows: 5 mg of the catalyst was added to 50 mL solution of a pollutant (50 ppm) in a 100 mL glass beaker, and the mixture was sonicated for 5 min and then stirred in the dark for 55 min to reach an adsorption equilibrium. PMS (0.3 g L^−1^) was then rapidly added to the mixture. At a given exposure interval, 1.5 mL of the solution was taken out and filtered through a 0.22 μm filter. The concentration of the pollutant in the filtrate was measured using the UV–Vis spectrophotometer or the HPLC spectrophotometer.

### Catalytic Degradation of BCC/BCH Membranes in a Flow-Bed System

After the BCC//BCH membrane (1 × 3 cm^2^) was put at the bottom of the reactor, the pollutant solution and the PMS solution were pumped through the reactor at different flow rates. At a given interval, 1 mL of the treated solution was taken out and the concentration of the pollutant in the solution was measured with the UV–Vis spectrophotometer or the HPLC spectrophotometer.

### Calculation

The evaporation rate (*v*) of the solar-driven evaporator was evaluated by the mass change of the evaporated water using Eq. (1):

1 $$v = {\text{d}}m/(S \times {\text{d}}t)$$

where *m* (g) is the mass of the water evaporated, *S* (m^2^) is the projected area of the sample directly exposed to the simulated sunlight, and *t* (h) is the exposure time. The solar-thermal conversion efficiency (*η*) was calculated with Eq. (2):

2 $$\eta = vH_{LV}/(C_{opt} \times P_{0} )$$

where $H_{LV}$ is the total enthalpy of sensible heat and phase change from liquid to water vapor, $C_{opt}$ is the optical concentration, and $P_{0}$ is the nominal solar irradiation value of 1 kW m^−2^. When calculating the efficiency, the evaporation rate of water in the dark was deducted to eliminate the influence of natural water evaporation (*v *= *v*_*light*_–*v*_*dark*_). The degradation rate (*DR*) and the degradation rate constant (*k*) were calculated with Eq. (3, 4):

3 $$DR = C_{t}/C_{0}$$

4 $$kt = \ln C_{0} /C_{t}$$

where $C_{0}$ is the initial pollutant concentration (ppm), and $C_{t}$ is the pollutant concentration (ppm) at different time periods, and *t* is the reaction time (min).

### Measurement and Calculation of Equivalent Vaporization Enthalpy

Because the water evaporation is driven by the identical energy input (*U*_*in*_), the equivalent enthalpy (*ΔH*_*e*_) of water evaporation under the same experimental conditions (at 25 °C, no solar light irradiation) can be obtained from Eq. (5):

5 $$U_{in} = \Delta H_{e} *v = \Delta H_{e}^{\prime } *v^{\prime }$$

where $\Delta H_{e}$ and $\Delta H_{e}^{\prime }$ are the evaporation enthalpies of pure water or the water in different systems, and ${\text{v}}$ and ${\text{v}}{\prime}$ denote the evaporation rates in dark.

## Results and Discussion

### Fabrication and Hierarchical Structure of Bilayer BCC//BCH Membrane

**Fig. 2:**
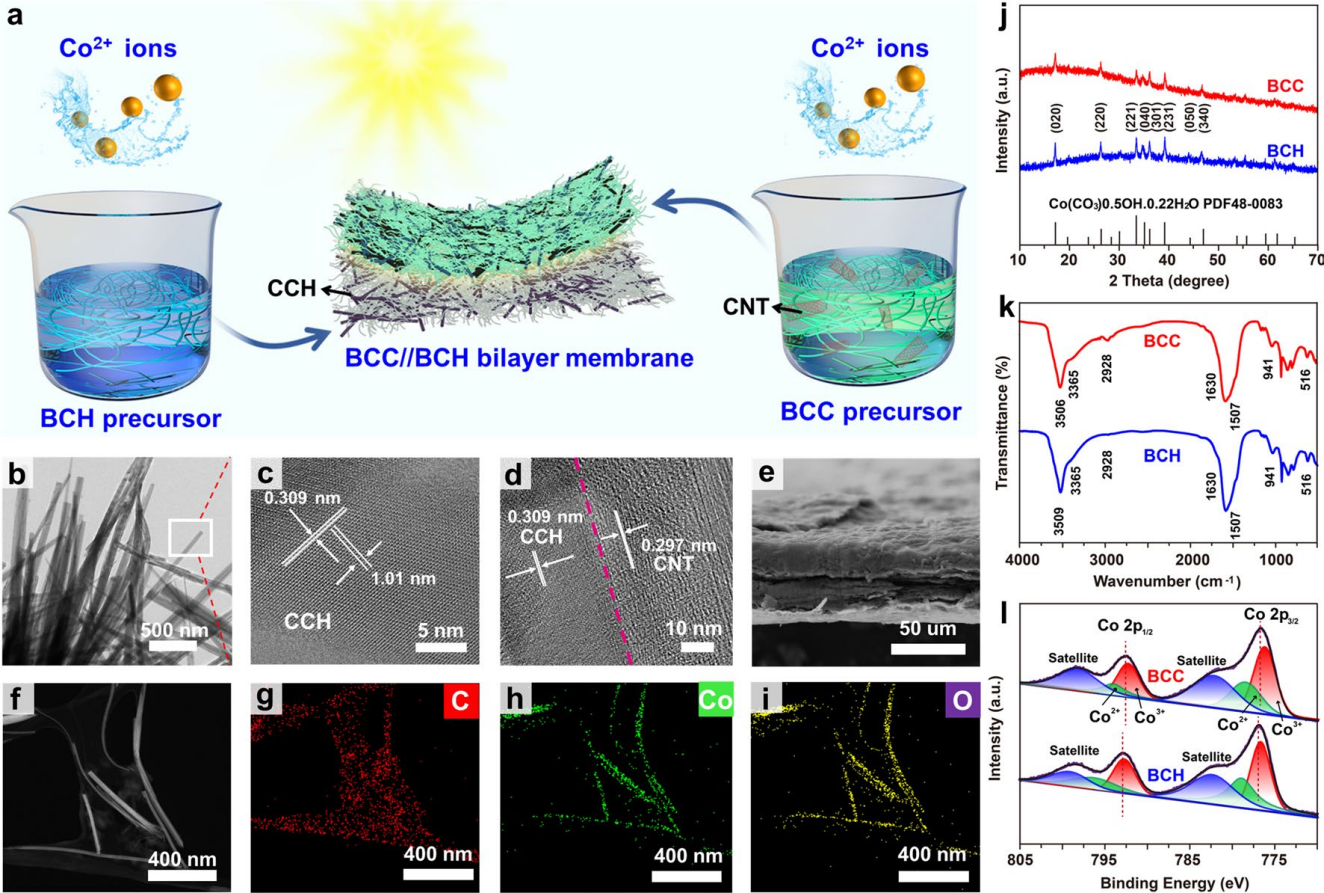
**a** Schematic illustration of preparing bilayer BCC//BCH membrane. **b** TEM and **c** HRTEM images of CCH nanorods. **d** HRTEM image of BCC hybrid. **e** Cross-sectional SEM image of BCC//BCH membrane. **f** HRTEM and **g-i** elemental mapping images of BCC. **j** XRD curves, **k** FT-IR spectra, and **l** Co 2*p* XPS spectra of BCC and BCH

The fabrication process of the bilayer BCC//BCH membrane is schematically illustrated in Fig. 2a. The decoration of CCH catalyst and solar-thermal CNTs on a hydrophilic BC substrate is achieved by in situ synthesis of CCH nanorods with an ethylene glycol-assisted hydrothermal process in the presence of the CNTs and BC components, obtaining the BCC layer, during which urea acts primarily as a precursor for the generation of hydroxyl and carbonate anions. CNTs are negatively charged with a zeta potential of – 40 mV [38] and used to enhance the solar light absorption capacity and modulate the hydrophobicity of the BCC layer. Over a wide pH range from 2 to 12, the surface charge of CCH is positive [39]. Obviously, the negative CNTs and the positive CCH nanorods can be readily assembled to fabricate the BCC layer. Similarly, the BCH layer is acquired by an in situ hydrothermal synthesis under the same conditions in the absence of CNTs (Fig. S1). The BCC//BCH bilayer membrane is manufactured by a layer-by-layer filtration approach. As shown in Fig. S2, the bottom BCH layer is light purple and still hydrophilic due to the hydrophilic BC substrate, facilitating the rapid upward transport of water, and the adsorption of contaminants. Meanwhile, the presence of CNTs gives the top BCC layer a dark appearance, enabling efficient solar light absorption and solar-thermal energy conversion, and makes the layer less hydrophilic to accelerate water evaporation and prevents salt deposition on the top surface of the membrane. Besides, the BCC//BCH membrane has a thickness of 46 μm (Fig. S2). The bare BC nanofibers, with diameters of 20–50 nm, exhibit randomly arranged fibrous structures after freeze-drying (Fig. S3), while the synthesized CCH presents a nanorod structure with a smooth and flat surface and a relatively uniform diameter distribution of 20–25 nm (Fig. 2b). The high-resolution transmission electron microscopy (HRTEM) image shows two sets of mutually perpendicular lattice fringes with interplanar distances of 0.309 and 1.01 nm, corresponding to the (300) and (010) crystal planes of the orthorhombic crystal (Fig. 2c) [40]. After integration with BC and CNTs, the CCH nanorods are uniformly anchored in the network of BC and CNTs, supporting each other to form a loose network with many voids (Fig. 2f). The (300) facets of the CCH are grown directly onto the CNTs without gaps (Figs. 2d and S4), resulting in tight contact for efficient heat transfer at the surface. Therefore, the dramatically elevated local solar-thermal temperature around the solar-thermal CNTs can directly boost the catalytic reaction of the CCH catalyst. The TEM image and corresponding elemental distribution maps of BCC show that the Co element derives mainly from CCH, while CCH, CNTs and BC all contain C and O elements (Fig. 2g–i). The asymmetric BCC//BCH membrane has an obvious boundary between the two layers (Fig. 2e).

The XRD patterns confirm the compositions of BCH and BCC (Fig. 2j). All diffraction peaks of BCH and BCC can be indexed to the monoclinic CCH rod (JCPDS card No. 48-0083) [40], while the vague and inconspicuous diffraction peak of BCC at ~ 26° corresponds to that of CNTs. The compositions of the as-prepared BCH and BCC layers are investigated with their FT-IR spectra in the range of 400–4000 cm^−1^ (Fig. 2k). The strong peaks at 3509 cm^−1^ for BCH and 3506 cm^−1^ for BCC are attributed to the stretching vibration of the O–H bond, whereas the peak at 3365 cm^−1^ for both BCH and BCC corresponds to the interaction between the carbonate anions and the O–H groups. The two peaks at 2928 and 1630 cm^−1^ of both BCH and BCC derive from the O–H stretching vibration and H–O–H bending vibrations of H_2_O, respectively, indicating the presence of crystal water in the as-prepared products [41]. The presence of CO_3_^2−^ in the products is evidenced by its mid- to lower-wave vibrational bands, suggesting the existence of polydentate or monodentate carbonate ligands. The other two characteristic peaks at 941 and 516 cm^−1^ are ascribed to the bending of Co–OH [42]. Compared to BCH [43], the Co 2*p* peaks of BCC are uniformly shifted to lower binding energies, suggesting an interaction between CCH and CNTs (Figs. 2l and S5).

### Hydrogen Bonding Networks and Evaporation Performances of the BCC//BCH Membrane

There are three types of water in the evaporation system: free water (FW), intermediate water (IW), and bound water (BW) [44]. The hydration layer with strong interaction on the surface of the evaporation system can be considered as the bound water, while the water close to the hydration layer with less interaction is the intermediate water. The intermediate water is activated water, and its evaporation efficiency is approximately 86 times that of free water [45]. Therefore, increasing the amount of intermediate water can effectively reduce the vaporization enthalpy of the water in the membrane and thus improve the water evaporation efficiency. Fortunately, the chemistry of the evaporation surface can influence the hydrogen bonding networks. As reported, the water in hydrogels can form strong hydrogen bonds with –OH groups of the hydrogel skeleton, promoting these water molecules to form weak hydrogen bonds with surrounding water molecules [46]. Besides, ions can be adsorbed on the evaporation surface, further affecting the hydrogen bonding at the catalyst surface. This means that creating abundant surface –OH groups or introducing proper ions can increase the amount of the intermediate water with weak hydrogen bonds.

**Fig. 3:**
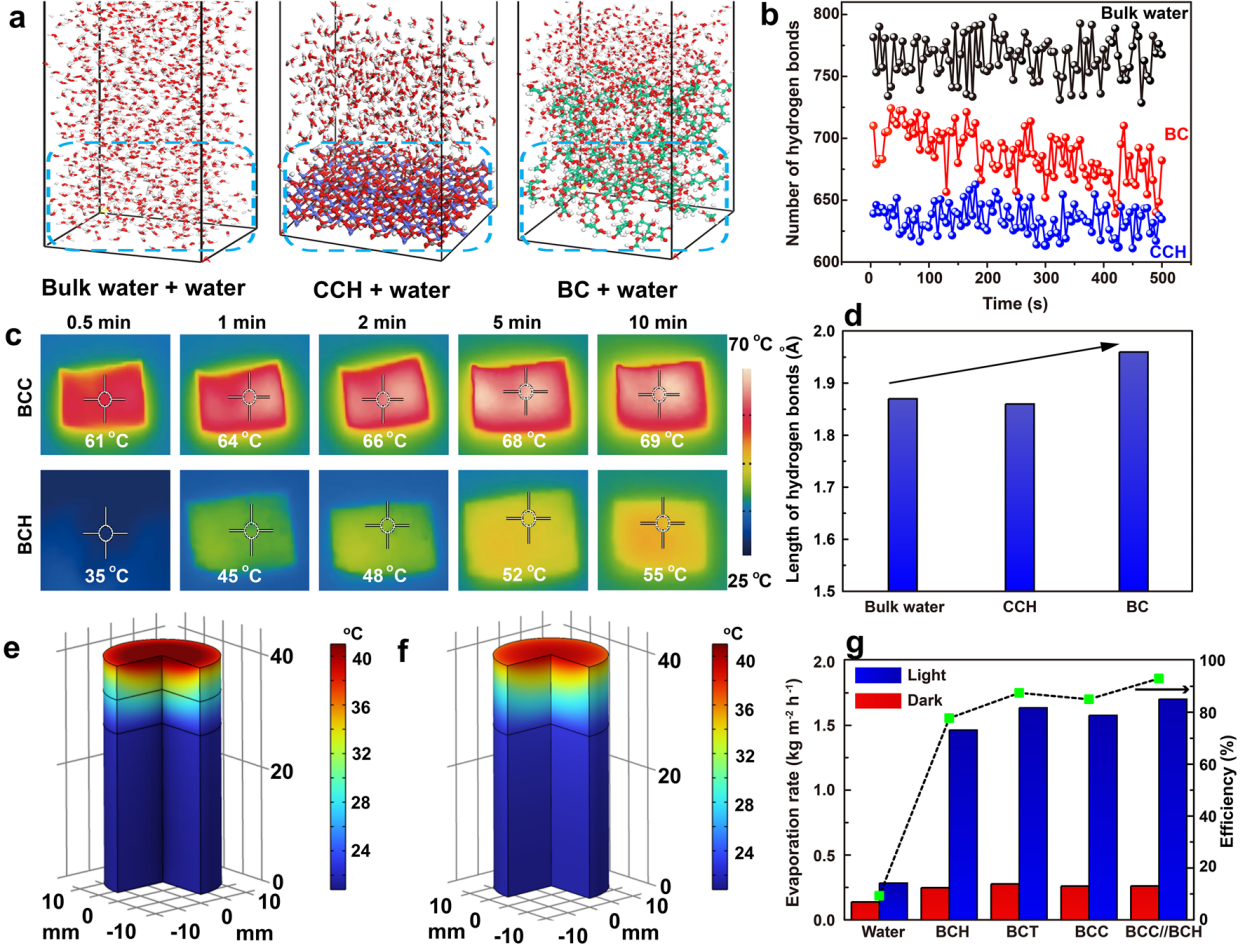
**a** MD simulations of bulk water, CCH-water, and BC-water units at 500 ps snapshots, and the corresponding **b** number and **d** length of hydrogen bonds with time. **c** Infrared images of BCC and BCH membranes in dry state. Simulation of the temperatures of **e** BCC//BCH and **f** BCC evaporators. **g** Comparison of solar steam generation rates and evaporation efficiencies of BCT, BCH, BCC, and BCC//BCH membranes under 1-sun irradiation and in the dark

Both BC and CCH possess abundant surface –OH groups and can form strong hydrogen bonds with surrounding water molecules, producing abundant intermediate water. As a result, the water equivalent enthalpy (*ΔH*_*e*_) of the BCC//BCH membrane in pure water is 1645 J g^−1^, much lower than 2430 J g^−1^ of bare water (Fig. S6). Furthermore, the molecular dynamics (MD) simulation results show that the strong interactions of water molecules with the CCH or BC strongly affect the hydrogen bonds between the water molecules themselves, resulting in a decrease in the number and strength of water–water hydrogen bonds (Fig. 3a, b, d). Weak hydrogen bonds can reduce the energy required to evaporate water. Therefore, the presence of CCH or BC components should result in a higher evaporation rate under the same solar energy input.

Furthermore, the solar steam generation performance of an evaporation device is strongly dependent on the solar light absorption efficiency and the solar-thermal energy conversion capacity of the solar-thermal materials. As shown in Fig. 3c, the surface temperature of the BCC membrane can quickly increase to 61 °C within 0.5 min under 1-sun irradiation, much faster than that of the BCH (35 °C). The BCC membrane eventually remains at a temperature of ~ 69 °C as the irradiation time increases to 10 min, demonstrating its remarkable solar-thermal conversion capability (Fig. S7a). Benefiting from the superb solar light absorption of CNTs in the entire solar spectral range, the BCC membrane exhibits an excellent solar light absorption of up to 96% in the solar spectral range of 250–2500 nm, far superior to ~ 58% of the BCH membrane (Fig. S7b). Even in wet state, the steady-state evaporation temperatures of BCT (the composition of BC and CNTs), BCC, and BCC//BCH membranes under 1-sun irradiation can reach 39.9, 41.0, and 41.8 °C, respectively, higher than 36.2 °C of the BCH without the presence of CNTs (Figs. S8, S9).

The hydrophilicity of the top and bottom layers of the BCC//BCH membrane also influences the solar-driven water evaporation performance. Although the upward transfer paths of water should have good hydrophilicity, it is better for the evaporation surface to exhibit a certain degree of hydrophobicity, because the hydrophilic surface could cause more heat to be lost to the water, thereby reducing the temperature of the evaporation surface and the corresponding evaporation rate. As shown in Fig. S10, the water droplet can be adsorbed within 15 s due to the hydrophilic nature of the bottom BCH layer, while the droplet on the top BCC layer still exhibits a water contact angle of 46^o^ even after 60 s, implying that the bottom BCH layer has a better hydrophilicity than the top BCC layer. The bottom hydrophilic BCH layer facilitates rapid upward transport of water, while the top layer of the less hydrophilic BCC can maintain a high surface temperature for boosting the water evaporation by decreasing the contact of the evaporation surface with the bulk water to some extent. Besides, the bottom BCH layer has a lower thermal conductivity than the top BCC layer (Fig. S11), indicating that the bottom BCH layer can provide the BCC//BCH membrane with a better thermal management capability than the BCC membrane alone.

The heat management capability is evaluated by simulating the temperature distribution of the evaporators. As depicted in Fig. 3e, f, the BCC//BCH membrane evaporator exhibits a higher surface temperature and a significant heat localization effect than its BCC counterpart. These results demonstrate that the design of the asymmetric membrane is effective in reducing the heat diffusion to the bulk water and promoting the solar-driven steam generation efficiency, confirming the effective thermal regulation of the BCC//BCH membrane. As a result, the BCC//BCH membrane-based solar-thermal water evaporator exhibits a high evaporation rate of 1.70 kg m^−2^ h^−1^ (Fig. S12), which is higher than that of pure water (0.28 kg m^−2^ h^−1^), BCH (1.46 kg m^−2^ h^−1^), BCC (1.58 kg m^−2^ h^−1^), and BCT (1.64 kg m^−2^ h^−1^). The corresponding solar-thermal conversion efficiencies of pure water, BCT, BCH, BCC, and BCC//BCH membranes are calculated to be 9.3%, 87.5%, 77.7%, 85.0%, and 93.0%, respectively (Fig. 3g), implying that both CNTs and CCH nanorods contribute to the solar-thermal energy conversion process. More interestingly, the asymmetric structure can further promote solar-thermal conversion, improving the solar-driven water evaporation rate. Under 3-sun downward irradiation, the BCC//BCH evaporator can reach a high water evaporation rate of 4.73 kg m^−2^ h^−1^ (Fig. S13). It indicates that the solar-thermal BCC//BCH membrane retains its excellent solar-thermal conversion capability for efficient water evaporation under varying solar radiation intensities, exhibiting prominent performances compared to relevant evaporators reported (Fig. S14, Table S1) [47–69].

### Catalytic Degradation Performances and Mechanisms of the BCC//BCH Membrane

**Fig. 4:**
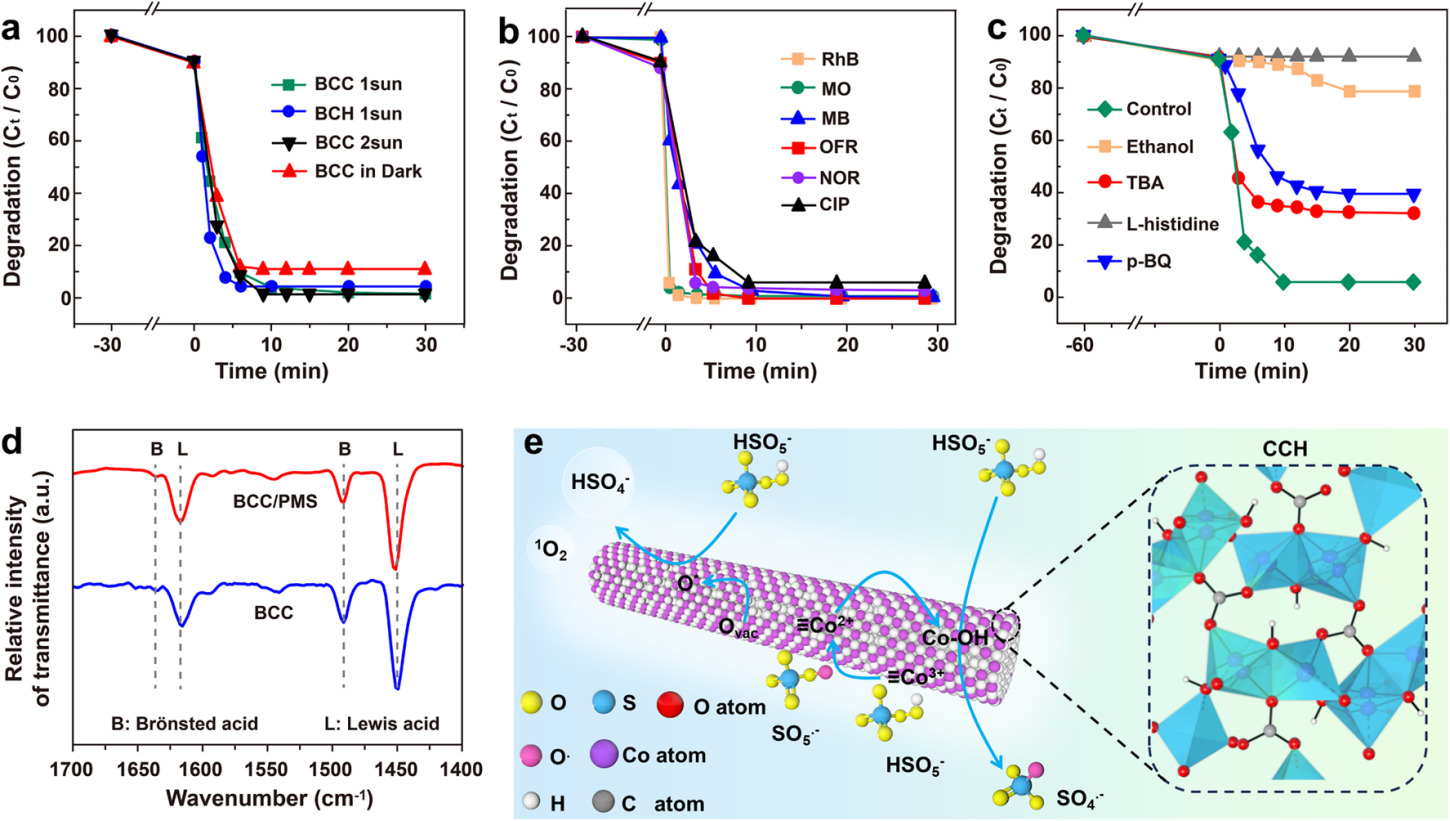
**a** Comparison of degradation efficiencies of CIP using different samples under different solar light irradiations. **b** Degradation performances of BCC powder toward dyes and antibiotics. **c** Effect of scavengers on CIP degradation by BCC. **d** Pyridine infrared spectra of BCC and BCC/PMS systems. **e** Schematic diagram of the catalytic reaction mechanisms of the BCC

The in situ synthesized CCH nanorods endow both BCC and BCH with highly efficient catalytic degradation capabilities, enabling them to degrade organic pollutants in bulk water during the solar steam generation process. As shown in Fig. 4a, both BCH and BCC can rapidly degrade CIP. The catalytic degradation efficiency of the BCC overperforms that of the BCH, obtaining almost 100% degradation of CIP. Interestingly, increasing the solar radiation intensity can accelerate the catalytic degradation of CIP with an apparent activation energy of 53.9 kJ mol^−1^ in the BCC/PMS system (Fig. S15). These results indicate that CNTs not only contribute to the solar light absorption and solar thermal energy conversion, but also promote the catalytic degradation via the solar excitation effect. Besides, the BCC/PMS system can catalytically degrade different dyes (RhB, MB and MO), antibiotics (OFL, NOR), and VOCs (phenol) effectively with high degradation rate constants within 3–10 min (Figs. 4b and S16), demonstrating its outstanding universality. Both the catalytic degradation and the evaporation performance are directly related to the free radicals and non-radical species generated by the activation of PMS in AOPs. Scavengers, such as L-histidine, ethanol (EtOH), _P_-benzoquinone (_P_-BQ), and tert-butanol (TBA), are selected to trap specific active species of ^1^O_2_, SO_4_^·−^, ^·^O_2_^−^, and ^·^OH during the catalytic degradation process, respectively. As shown in Fig. 4c, the addition of L-histidine, EtOH, _P_-BQ, and TBA to the BCC/PMS system can reduce the degradation rate of CIP to 7.5%, 21.2%, 60.7%, and 67.7%, respectively, and their reaction rate constants decrease accordingly with the same orders (Fig. S17c). It confirms that ^1^O_2_ is the main active agent in the CIP degradation process, SO_4_^·−^ plays a secondary role, and the contribution of ^·^O_2_^−^/^·^OH is less significant, which can be supported by the electron paramagnetic resonance (EPR) results (Fig. S18).

As a highly efficient and environmentally friendly oxidant, the ^1^O_2_ in the excited state of molecular oxygen can derive from oxygen vacancies (O_vac_) or ≡Co–OH^+^ on the catalyst surface or from liquid phase reactions [70, 72]. As shown in Fig. S19, there are a large number of O_vac_ on the surface of the BCC catalyst. Owing to the high energy of the defect sites, the O_vac_ can easily anchor other heteroatoms or groups to form new active centers. On the one hand, O_vac_ can enhance the mobility of the surrounding oxygen ions, thus contributing to the generation of reactive oxygen species (Reactions 6–8), where O_o_^×^ refers to the oxygen ions located in O_vac_ [71]:

6 $$\equiv {\text{Co}}^{2 + } + {\text{HSO}}_{{5}}^{ - } + {\text{H}}_{2} {\text{O}} + {\text{O}}_{{{\text{vac}}}} \to\; \equiv {\text{Co}}^{3 + } + \cdot {\text{OH}} + {\text{SO}}_{4}^{2 - } + 2{\text{H}}^{ + } + {\text{O}}_{{\text{o}}}^{ \times }$$

7 $$\equiv {\text{Co}}^{{{2} + }} + {\text{HSO}}_{{5}}^{ - } + {\text{H}}_{{2}} {\text{O}} + {\text{O}}_{{{\text{vac}}}} \to \;\equiv {\text{Co}}^{{{3} + }} + {\text{H}}^{ + } + {\text{SO}}_{{4}}^{ \cdot - } + {\text{ O}}_{{\text{o}}}^{ \times }$$

8 $$\equiv {\text{Co}}^{{{3} + }} + {\text{HSO}}_{{5}}^{ - } + {\text{H}}^{ + } + {\text{O}}_{{\text{o}}}^{ \times } \to\; \equiv {\text{Co}}^{{{2} + }} + {\text{SO}}_{{5}}^{ \cdot - } + {\text{H}}_{{2}} {\text{O}} + {\text{O}}_{{{\text{vac}}}}$$

On the other hand, a faster conversion between Co^2+^/Co^3+^ redox pairs can be realized via an efficient charge transfer promoted by the O_vac_, and the generation of ^1^O_2_ for the degradation of organic pollutants can be achieved effectively (Reactions 9–11), where O^*^ is the activated adsorptive oxygen [72]:

9 $$\equiv {\text{2Co}}^{{{3} + }} + {\text{O}}^{{{2} - }} \to \equiv {\text{2Co}}^{{{2} + }} + {\text{O}}_{{{\text{vac}}}} + 0.{\text{5O}}_{{2}}$$

10 $${\text{O}}_{{{\text{vac}}}} \to {\text{O}}^{*}$$

11 $${\text{O}}^{*} + {\text{HSO}}_{{5}}^{ - } \to {\text{HSO}}_{{4}}^{ - } +^{{1}} {\text{O}}_{{2}}$$

Particularly, there is no obvious deviation and intensity attenuation in the XPS spectra of the used BCC catalyst (Fig. S20a), confirming the satisfactory structural stability of BCC. In addition, the Co^2+^/Co^3+^ ratio increases from 98 to 99% after the catalytic reaction (Fig. S20b), which might be attributed to the increased number of O_vac_ after the reaction, which reduces the chemical valence of the Co cations by creating electron-rich regions and increasing the density of oxygen ions, thereby leading to the faster conversion between Co^2+^/Co^3+^ redox pairs, further providing greater electron transport capacity for the activation of PMS.

The first step of the Co-based catalyst for the PMS activation is the formation of surface cobalt hydroxyl groups (≡Co–OH^+^) in the aqueous solution, which is a rate-limiting step. The catalyst with abundant surface cobalt hydroxyl groups is superior to commercial cobalt oxide catalysts [73]. The existence of ≡Co–OH^+^ on BCC can be proved by the pyridine-FT-IR results. As shown in Fig. 4d, the transmission bands at 1448 and 1616 cm^−1^ can be assigned to the pyridine adsorbed on the Lewis acid sites, while the peaks at 1491 and 1635 cm^−1^ are attributed to the Brønsted acid sites [74]. For the BCC sample, the peak intensities of the Lewis acid sites are much higher than those of the Brønsted acids, suggesting that the Lewis acids, such as the surface hydroxyl groups, are the dominant acidic sites for PMS adsorption and activation, which is consistent with the FT-IR results (Fig. 2k) and further confirms the presence of ≡Co–OH^+^ on the BCC surface. Moreover, the molar ratio of Brønsted acids to Lewis acid (B/L) increases from 0.11 to 0.14 after the adsorption of PMS on the BCC catalyst, demonstrating that the Lewis acid sites are responsible for the activation of PMS during the catalytic degradation process to generate various reactive oxygen species for promoting the catalytic degradation performances. Finally, all of these reactive oxygen species can effectively oxidize the CIP molecules to intermediates and further degrade the intermediates to CO_2_ and H_2_O (Fig. 4e).

### Regulation of Hydrogen Bonding Networks and Solvated Structures During the Evaporation and Catalytic Degradation Process

**Fig. 5:**
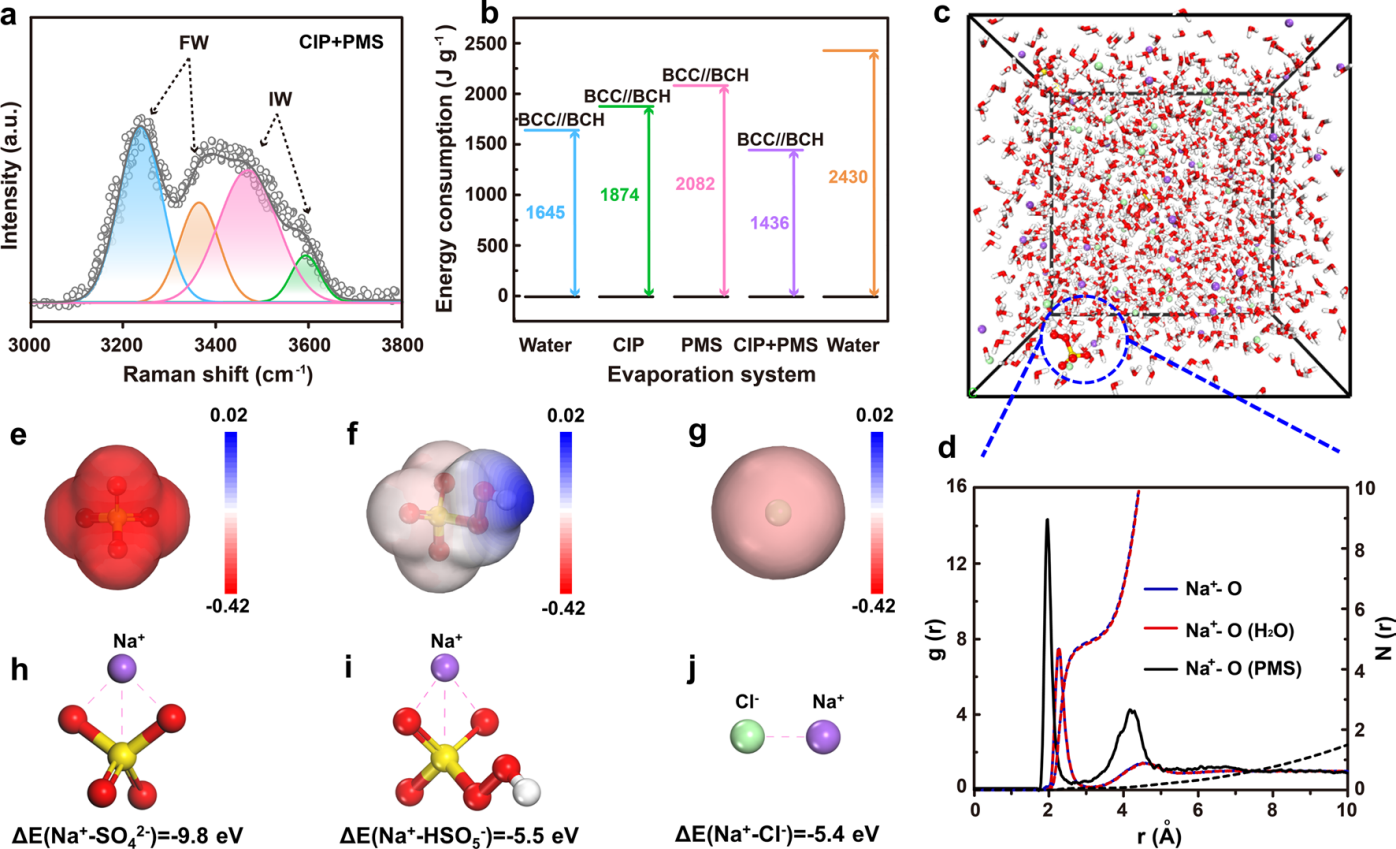
**a** Fitting curve in the energy region of the O–H stretching mode of water in the BCC//BCH-PMS-CIP system. **b** Vaporization enthalpy of water in different evaporation systems. **c** MD simulation snapshot and the corresponding **d** radial distribution functions profiles and coordination numbers of Na^+^. **e**–**g** ESP maps showing the charge distributions of solvent molecules and **h**–**j** their interaction energy with Na^+^

During the catalytic degradation process, hydrogen bonding networks that are highly related to the solar-driven water evaporation efficiency are affected by the dynamically generated ions (such as SO_4_^2−^ and HSO_5_^−^) and radicals. These ions and radicals derived from the catalysis process can solvate surrounding water molecules, in turn create more intermediate water (Fig. S21). Based on the Raman spectra, the IW/FW ratios for pure water, CIP, PMS and CIP-PMS systems are 0.32, 0.26, 0.36, and 0.96, respectively (Figs. 5a and S22), confirming that the AOPs can increase the proportion of intermediate water. As a result, the *ΔHe* of the BCC//BCH membrane in the presence of CIP and PMS is 1436 J g^−1^, which is even lower than 1645 J g^−1^ of the BCC//BCH membrane in pure water (Figs. 5b and S23).

As displayed in the MD simulation of various systems, the introduction of ions and radicals during the catalytic process promotes the hydration chemistry of BC [75]. For simplicity, we construct a molecular structure of BC containing two chains in different systems. It is seen that the ions and free radicals produced during the catalysis are capable of weakening the hydrogen bonds of BC molecules. As shown in Fig. S24, the average number of H-bonds decreases with the introduction of various ions and radicals generated in the catalyst process, indicating the weak interaction between BCs. Therefore, it is inferred that the ions and radicals generated during the catalyst process would break the hydrogen bonds between the hydroxyl groups. The snapshot from the MD simulation and corresponding radial distribution functions (RDFs) results reveal that the average coordination number of the O atom in the Na^+^ solvation shell is ~ 4.95, while the average coordination number of H_2_O in the Na^+^ solvation shell is ~ 4.89 in the presence of PMS (Fig. 5c, d), indicating that PMS is involved in the solvated shell layer of Na^+^. Furthermore, the O atoms in SO_4_^2−^ and HSO_5_^−^ show more negative charge densities than Cl atoms in the electrostatic potential (ESP) maps, suggesting that SO_4_^2−^ and HSO_5_^−^ can occupy the solvation shell of Na^+^ via the interaction of Na and O, which is confirmed by the density functional theory (DFT) calculations and molecular dynamics (MD) simulations (Fig. 5e–g). The binding energy between Na^+^ and SO_4_^2−^ is − 9.8 eV, higher than that of Na^+^/HSO_5_^−^ (− 5.5 eV) and Na^+^/Cl^−^ (− 5.4 eV), indicating that the combination between Na^+^ and SO_4_^2−^ is more feasible (Fig. 5h–j). More importantly, the higher binding energy of Na^+^ and SO_4_^2−^ could hinder the de-solvation of solvated Na^+^, hence preventing the deposition of NaCl.

**Fig. 6:**
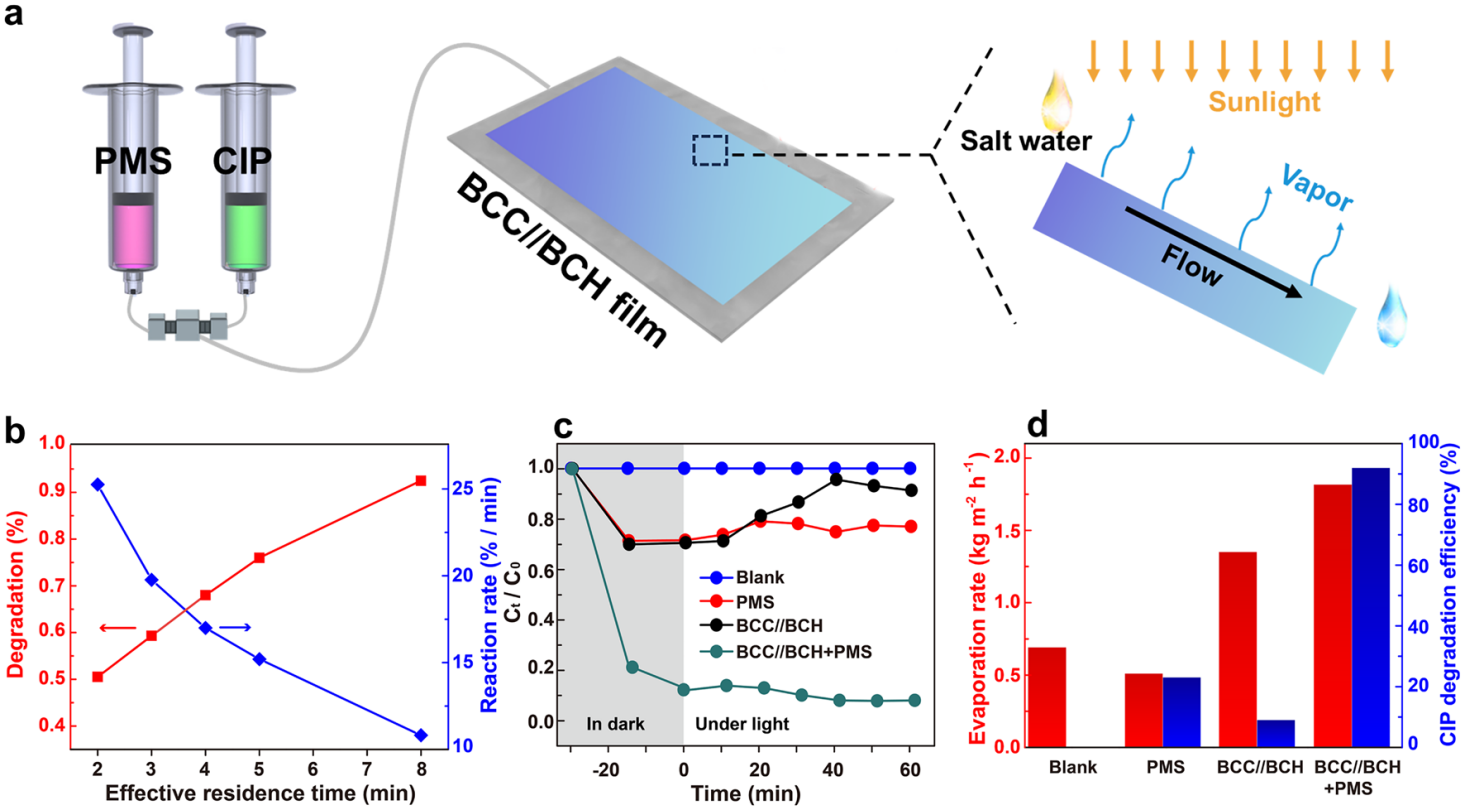
**a** Diagram of the flow-bed device for simultaneous solar steam generation and catalyst degradation. **b** Plots of degradation efficiency and reaction rate of the CIP pollutant at different flow rates. **c** Plots of CIP degradation evolution and **d** water evaporation rate under controlled conditions

Furthermore, a flow-bed water purification system is established for practical application (Fig. 6a) by taking advantage of the regulation of hydrogen bonding networks and solvation structures, the high solar light absorption capacity, the outstanding catalytic degradation effect, and the effective heat and water transport of the bilayer BCC//BCH membrane. The BCC//BCH membrane is placed at the bottom of the home-made flow-bed system. The CIP and PMS solutions are mixed via a three-way interface and injected into one end of the flow-bed system at a constant flow rate under the action of a peristaltic pump. As the CIP and PMS solution continuously flows through the BCC//BCH membrane, water is solar-heated and continuously evaporated to produce clean water vapor on the basis of the solar-thermal conversion effect of the BCC//BCH membrane. Meanwhile, the BCC//BCH membrane with the PMS can effectively degrade the CIP contaminant, and allow the purified water to be collected at the other end of the flow-bed system. The rich surface –OH groups of the BC and CCH (Fig. 3d) and the hydration effect of the dynamically AOP-generated anions (such as SO_4_^2−^ and HSO_5_^−^) and radicals can effectively regulate the hydrogen bonding networks to obtain more intermediate water for benefiting the solar steam generation. As a result, the evaporation performance can be further promoted regardless of increasing the amounts of catalyst or PMS (Figs. S25 and S26). Obviously, the flux rate influences the catalytic activity and water evaporation performance, because it affects not only the residence time of the CIP pollutant in the flow-bed system but also the exposure time of the water to solar light (Fig. S27). As the flow rate increases, the CIP degradation efficiency of the flow-bed system gradually decreases from ~ 92.0% at 10 mL h^−1^ to ~ 55.5% at 30 mL h^−1^ (Fig. 6b). It is noted that, at high flow rates, the pollutant has no sufficient time to contact the catalyst and be degraded. Although the reduction of the flow rate to 5 mL h^−1^ could further enhance the degradation efficiency of CIP to 99.2% (Figs. S28–S30), the flow rate of 10 mL h^−1^ is chosen as the flow rate for subsequent measurements after considering the high CIP degradation efficiency and the short retention time.

As shown in Fig. 6c, the BCC//BCH flow-bed purification evaporator presents a negligible adsorption effect on CIP, as the relative concentration (C_t_/C_0_) of the CIP solution decreases by only 8.6% (BCC//BCH). When exposed to 1-sun irradiation for 60 min, CIP is barely degraded (blank), and only a small proportion of 23.0% CIP is eliminated by PMS. In contrast, after combining PMS with the BCC//BCH membrane under 1-sun irradiation, 92.0% of CIP is removed, demonstrating the apparent catalytic effect of CCH in activating PMS. Interestingly, the BCC//BCH system with both PMS and CIP delivers a high evaporation rate of 1.81 kg m^−2^ h^−1^, superior to that of the BCC//BCH counterpart (1.40 kg m^−2^ h^−1^) (Fig. 6d). Furthermore, it also confirms that the AOPs can promote the water evaporation performance via the hydration effect of the AOP-generatedions and radicals.

### Salt Resistance Performances and Mechanisms of the BCC//BCH Membrane

**Fig. 7:**
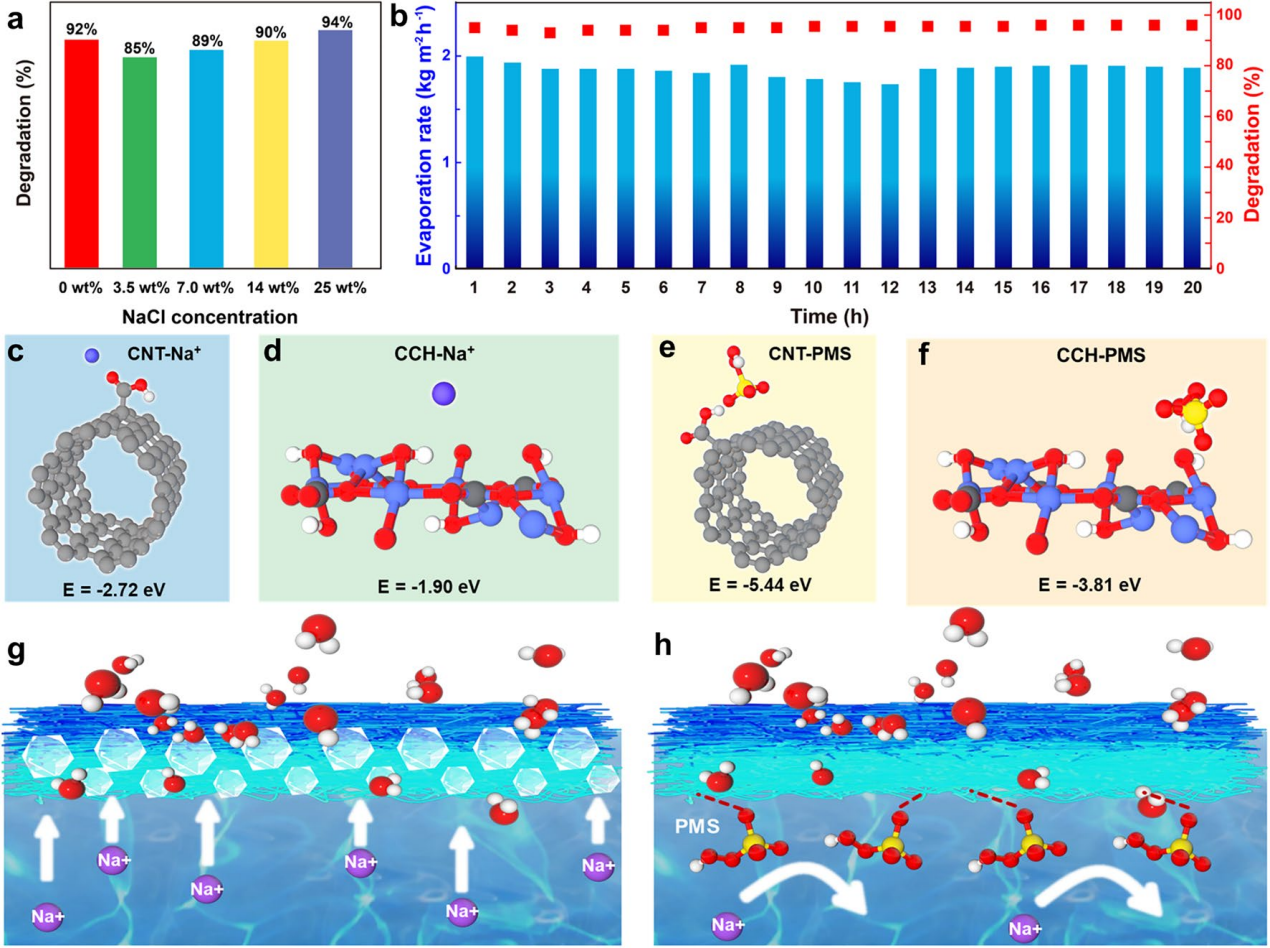
**a** Flow-bed catalytic degradation efficiencies at different NaCl concentrations. **b** Durability test of the flow-bed system to purify wastewater with 25 wt% NaCl and 50 ppm CIP under 1-sun irradiation. Molecular models of interactions between **c** CNT-COOH and Na^+^ ion; **d** CCH and Na^+^; **e** CNT-COOH and PMS; and **f** CCH and PMS. Schematic illustration of two types of situations during the solar desalination process: **g** without and **h** with the AOPs

For continuous purification of complex wastewaters containing both organic pollutants and a large number of salt ions, it is necessary to ensure the simultaneous catalytic degradation and solar-thermal water evaporation efficiencies of the flow-bed purification system. With increasing the NaCl concentration in the CIP effluents to 3.5, 7.0, and 14 wt%, the flow-bed system exhibits the CIP degradation efficiencies of 85%, 89%, and 90%, respectively, slightly lower than the 92% of CIP degradation efficiency in the absence of the NaCl, which is because Cl^−^ can react with HSO_5_^−^ and SO_4_^·−^/^·^OH in the PMS system to form Cl^·^ and other chlorinated compounds (HOCl, Cl_2_^·−^) with slightly lower activities than SO_4_^·−^ and ^·^OH, resulting in the slightly reduced degradation efficiency. Interestingly, when the concentration of NaCl in the system is saturated, a higher CIP degradation rate of 94% is achieved (Fig. 7a). Although the chlorine-containing active species have slightly lower activities than SO_4_^−^ and ^.^OH, the amount of chlorine-containing active species generated at the high Cl^−^ concentrations is large enough to degrade the CIP pollutant rapidly. These results indicate that the flow-bed purification system is well suited for catalytic degradation of contaminants at high salt concentrations, exhibiting stable water evaporation rates of ~ 1.88 kg m^−2^ h^−1^ and the CIP degradation efficiency of ~ 94% for at least 20 h for purifying wastewater containing 25 wt% NaCl and 50 ppm CIP (Fig. 7b), demonstrating that the designed flow-bed purifying system can continuously and simultaneously purify wastewater containing both organic pollutants and salt ions.

Deposition of NaCl on the evaporation surface involves three steps: de-solvation of solvated Na^+^, nucleation of NaCl, and growth of NaCl. The resistance to NaCl is attributed to the regulation of the Na^+^ solvation structure and the prevention of NaCl nucleation. As discussed in Fig. 5, the high binding energy of Na^+^ and SO_4_^2−^ can prevent the de-solvation of solvated Na^+^, thereby preventing the deposition of NaCl. Furthermore, during the solar-thermal desalination process, the continuous flowing can effectively hinder the salt deposition by preventing the nucleation and growth of salts [76]. Moreover, the nucleation of NaCl is also prevented during the catalytic degradation process. As shown in Fig. 7c–f, the interaction energies of PMS with CNT and CCH are  − 5.44 and − 3.81 eV, respectively, much stronger than those of Na^+^ with CNT (− 2.72 eV) and CCH (− 1.90 eV), implying that PMS is preferentially adsorbed on the evaporation surface and occupies the adsorption sites of Na^+^, thus preventing the salt crystallization on the evaporation surface effectively and efficiently (Fig. 7g, h). Therefore, the salt crystals are not observed in the interlayer of the membrane during the catalytic degradation process (Fig. S31), although, in the absence of the AOPs, visible salt crystals appear in the BCC//BCH interlayers at the end of the evaporation process (Fig. S32). Additionally, the size of the salt crystals collected at the edge of the BCC//BCH membrane during the solar-thermal evaporation of the brine in the AOPs is about 44 nm, while the size of the salt crystals obtained in the same system in the absence of AOPs is slightly smaller (~ 39 nm) (Figs. S33–S35). Clearly, the AOP catalytic degradation process not only alleviates the salt crystallization and achieves the stable evaporation performances, but also affects the size and purity of the salt crystals, resulting in higher purity and larger grain sizes that could be easily collected during the water purification process.

## Conclusions

Dynamic regulations of hydrogen bonding network and solvation structure for synergistic solar-thermal desalination of seawater/brine and catalytic degradation of organic pollutants are demonstrated by designing a flow-bed water purification system with an asymmetric BCC//BCH bilayer membrane. In the solar-thermal evaporation and catalysis membrane, the rich surface –OH groups of BC and CCH can regulate the hydrogen bonding network to generate more intermediate water for improving solar-thermal evaporation of water. The dynamically generated ions and radicals derived from the AOPs can involve the solvation shell of Na^+^, further increasing the proportion of intermediate water and reducing the water vaporization enthalpy for better evaporation performances. Moreover, the strong interaction between SO_4_^2−^/HSO_5_^−^ and Na^+^ in the solvation structure of Na^+^ hinders the de-solvation of the solvated Na^+^, and the SO_4_^2−^ and HSO_5_^−^ can be preferentially adsorbed on the evaporation surface, which hinders the NaCl nucleation and growth process, resulting in excellent desalination performances. The CCH nanorods with rich surface ≡Co–OH^+^ and oxygen vacancies can shorten the activation time of PMS and accelerate the generation of free radicals (SO_4_^·−^, ^·^OH) and ^1^O_2_. These highly active species ultimately enable the removal of a wide range of organic pollutants. The flow-bed water purification system with the bilayer BCC//BCH membrane achieves a superior evaporation rate of 1.81 kg m^−2^ h^−1^. Even used to purify highly concentrated saline wastewater with 25 wt% NaCl and 50 ppm CIP, the flow-bed water purification system still achieves a rapid water evaporation rate of 1.88 kg m^−2^ h^−1^ and a high CIP degradation efficiency of 94% under 1-sun irradiation for at least 20 h. The work provides new insights for designing a novel solar-thermal water purification system for generating clean water via regulating hydrogen bonding networks and solvation structures.

## Supplementary Information

Below is the link to the electronic supplementary material.

Supplementary file1 (DOCX 10800 KB)
