# Supplementary material for: Dynamic Regulation of Hydrogen Bonding Networks and Solvation Structures for Synergistic Solar-Thermal Desalination of Seawater and Catalytic Degradation of Organic Pollutants
Source: Nanomicro Lett. 2024 Oct 23;17:48. doi: 10.1007/s40820-024-01544-9 (PMC11499520; doi:10.1007/s40820-024-01544-9)
Supplement: Supplementary file 1 — Supplementary file1 (DOCX 10800 KB) [file 40820_2024_1544_MOESM1_ESM.docx]

Supporting Information for

**Dynamic Regulation of Hydrogen Bonding Networks and Solvation Structures for Synergistic Solar-Thermal Desalination of Seawater and Catalytic Degradation of Organic Pollutants**

Ming-Yuan Yu^1^, Jing Wu^1,^*, Guang Yin^1^, Fan-Zhen Jiao^2^, Zhong-Zhen Yu^2,^*, Jin Qu^1,^*

^1^ Beijing Key Laboratory of Advanced Functional Polymer Composites, Beijing University of Chemical Technology, Beijing 100029, P. R. China

^2^ State Key Laboratory of Organic-Inorganic Composites, College of Materials Science and Engineering, Beijing University of Chemical Technology, Beijing 100029, P. R. China

*Corresponding authors. E-mail: [wuj@buct.edu.cn](mailto:wuj@buct.edu.cn) (Jing Wu); [yuzz@mail.buct.edu.cn](mailto:yuzz@mail.buct.edu.cn) (Zhong-Zhen Yu); [qujin@mail.buct.edu.cn](mailto:qujin@mail.buct.edu.cn) (Jin Qu)

**Supplementary Figures and Table**


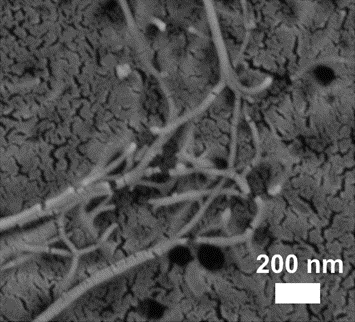


**Fig. S1** SEM image of BCH


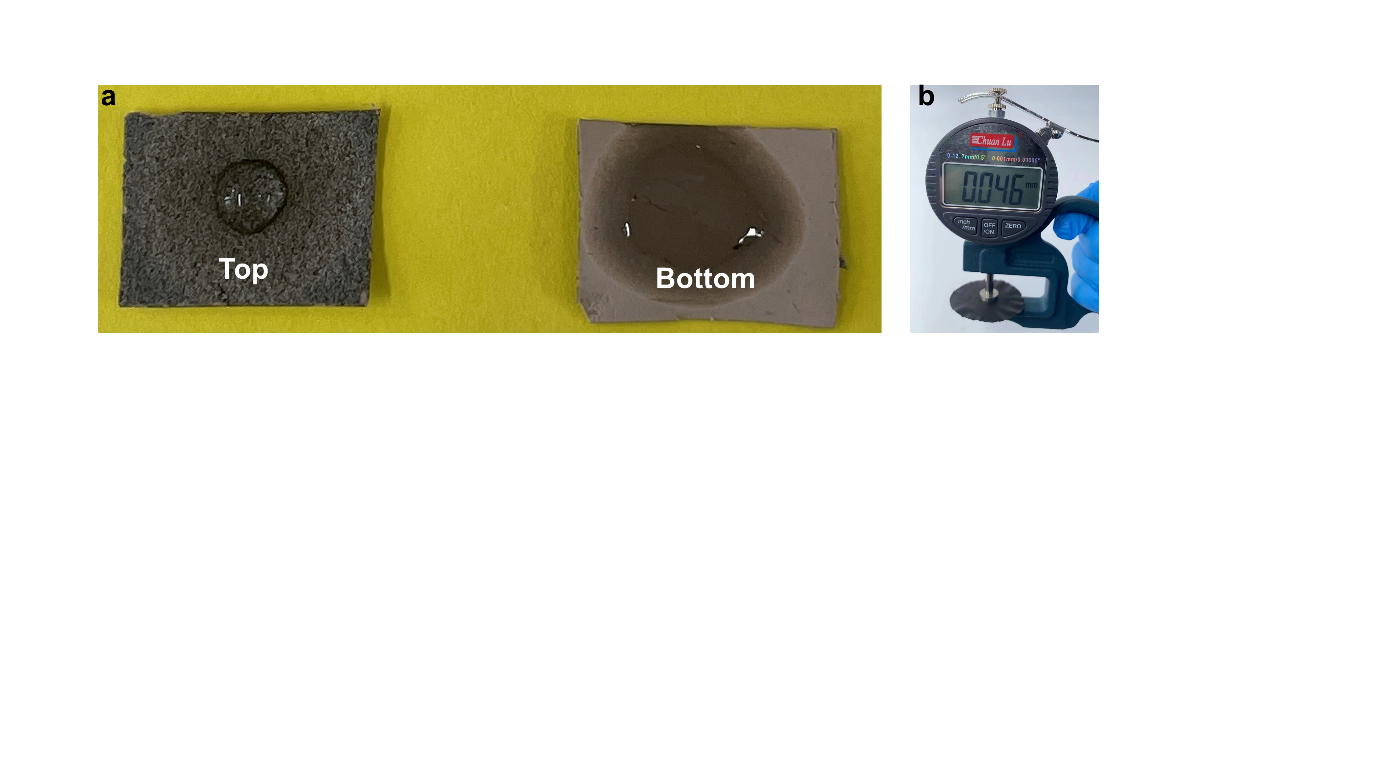


**Fig. S2** **a** Appearance and **b** thickness of the BCC/BCH membrane


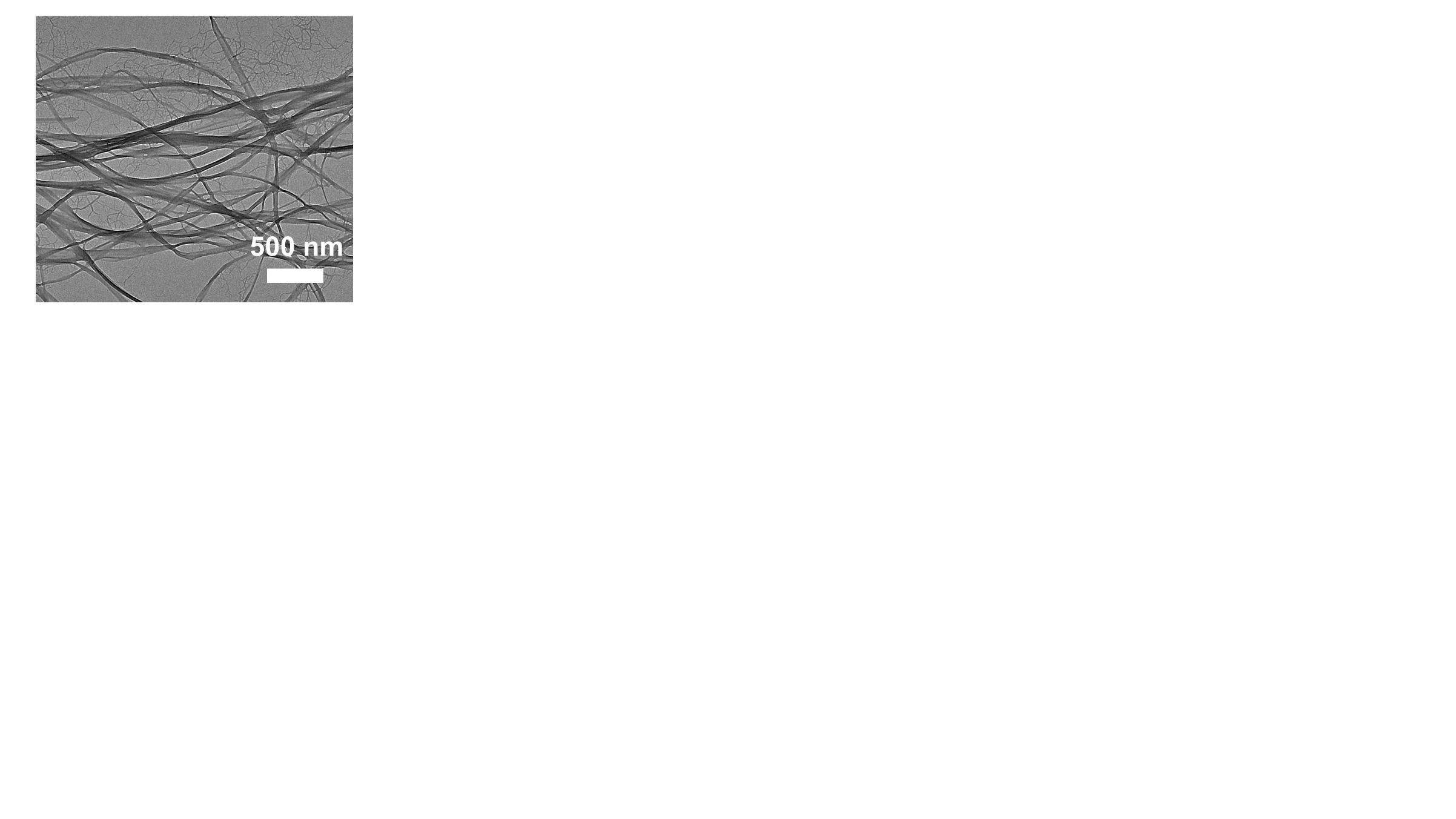


**Fig. S3** TEM image of bare BC


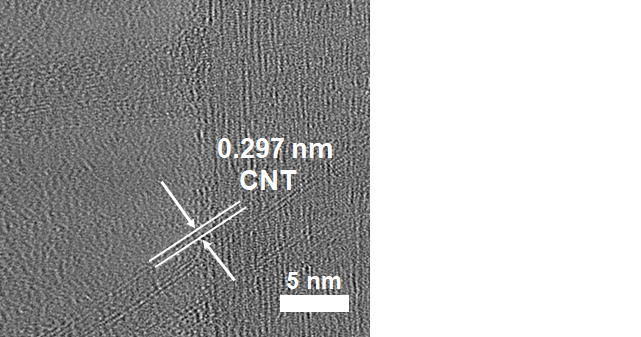


**Fig. S4** HRTEM image of CNTs


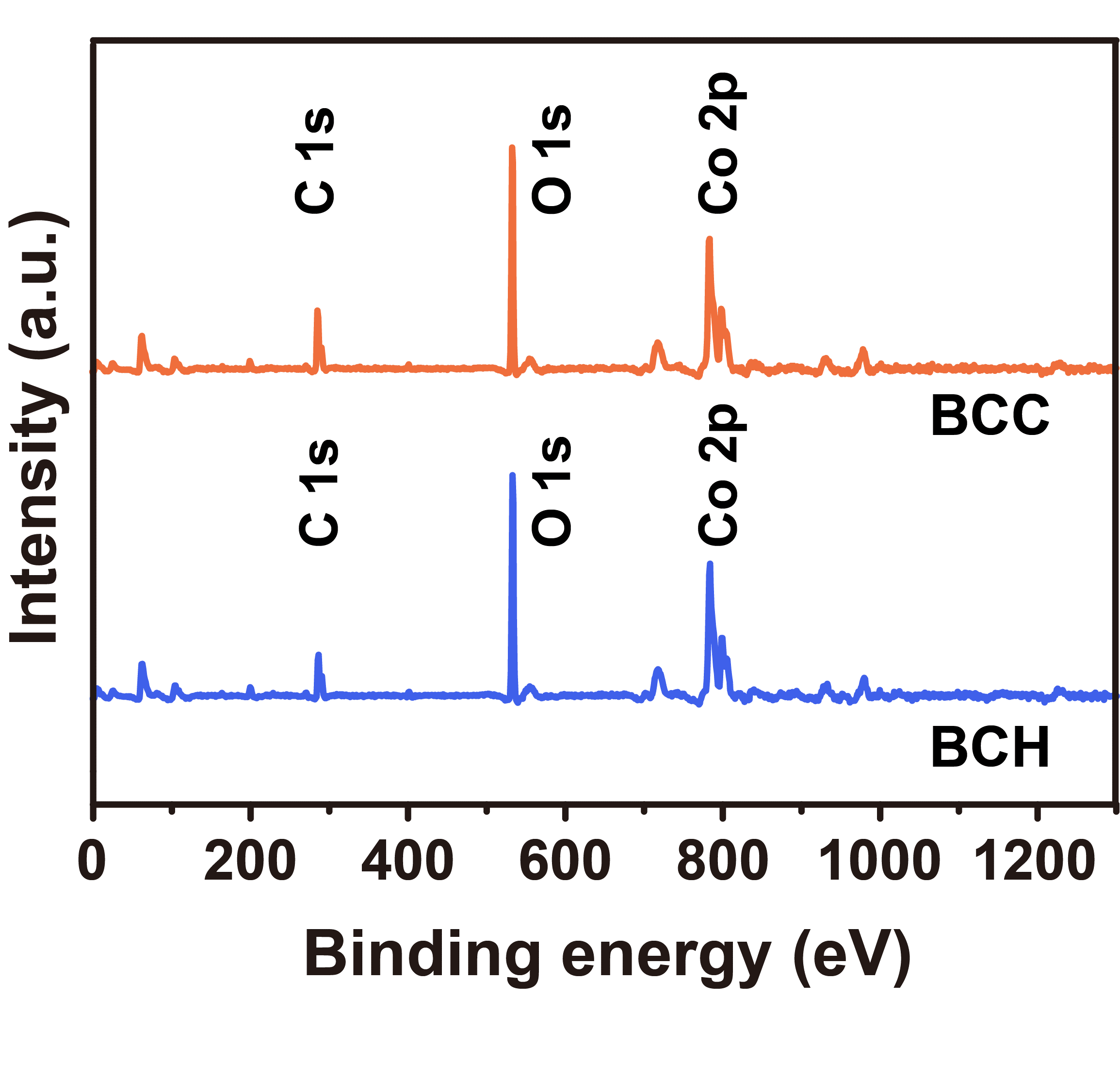


**Fig. S5** XPS spectrum of BCC and BCH samples


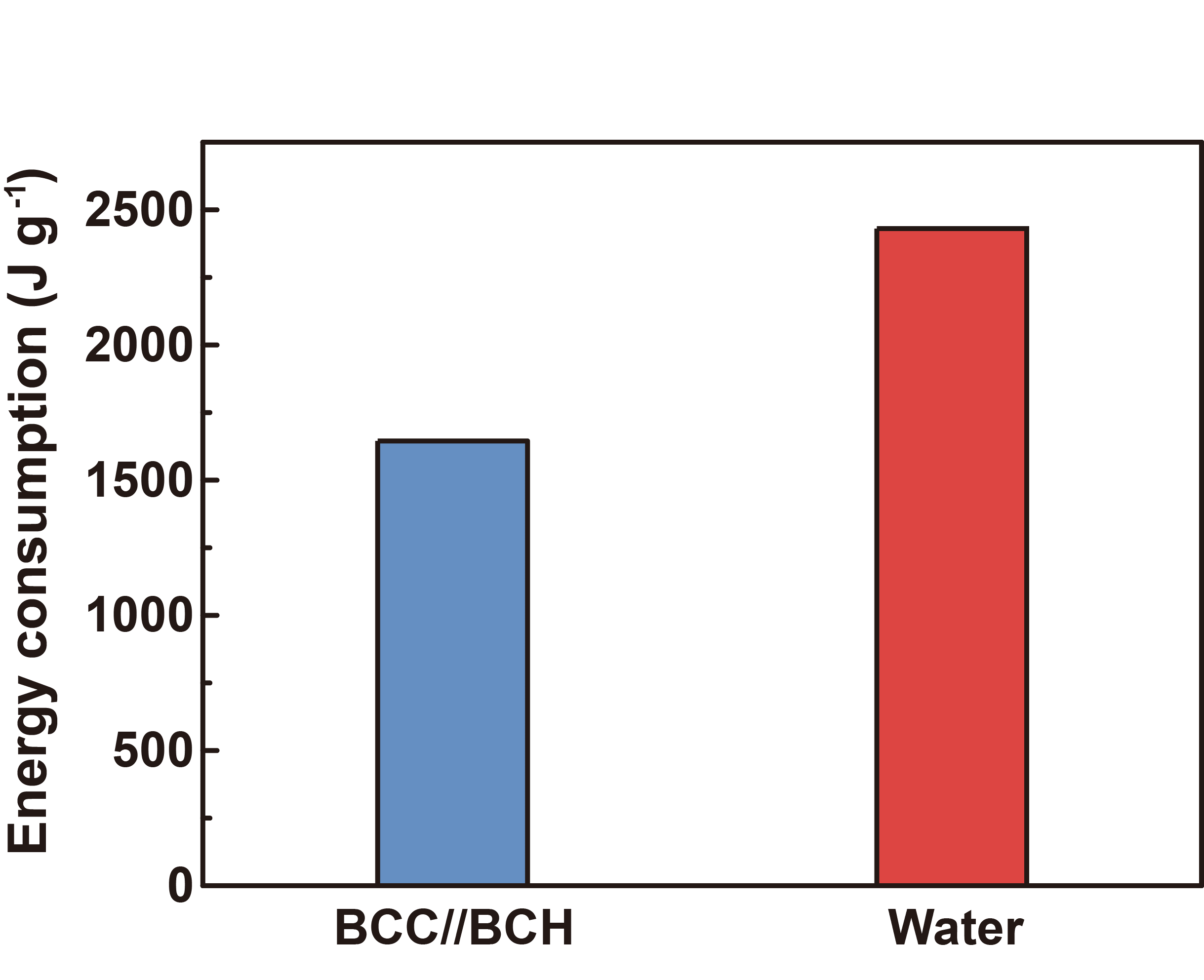


**Fig. S6** Evaporation enthalpy of water in different evaporation systems


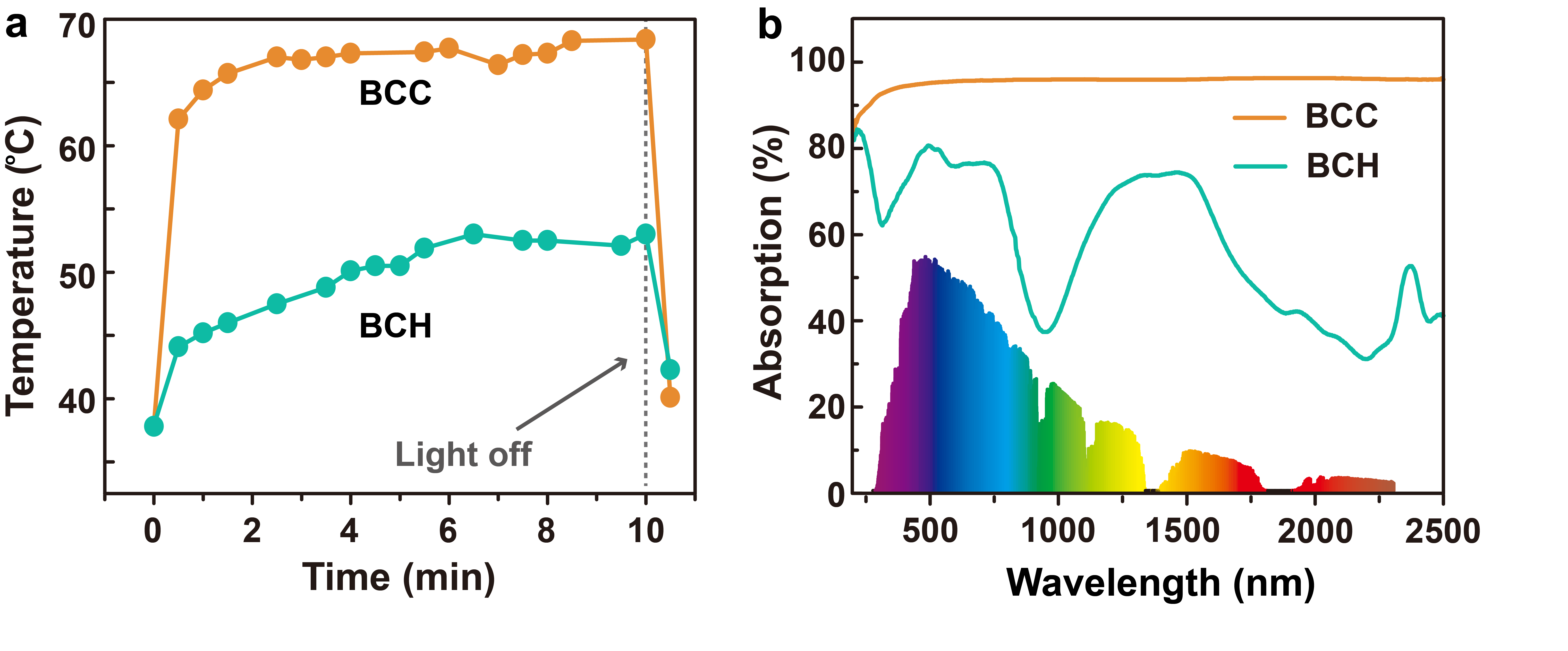


**Fig. S7** **a** Surface temperature of dry BCC and BCH membranes under 1-sun irradiation. **b** UV-Vis-NIR absorption spectra of BCH and BCC membranes


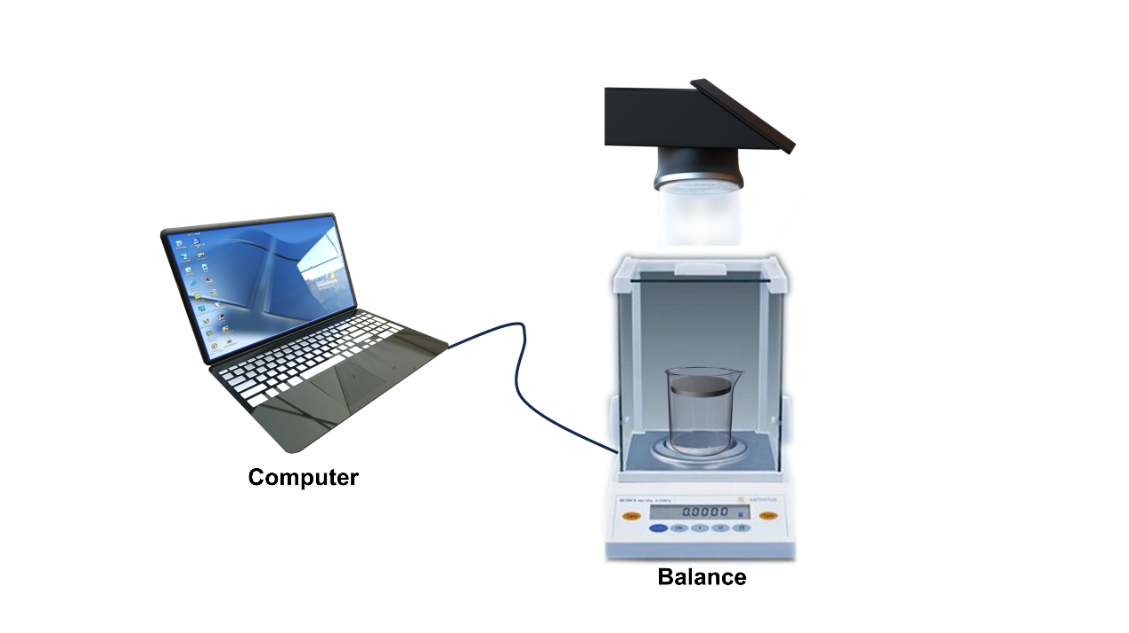


**Fig. S8** Schematic illustration of the water evaporation measurement under downward solar light


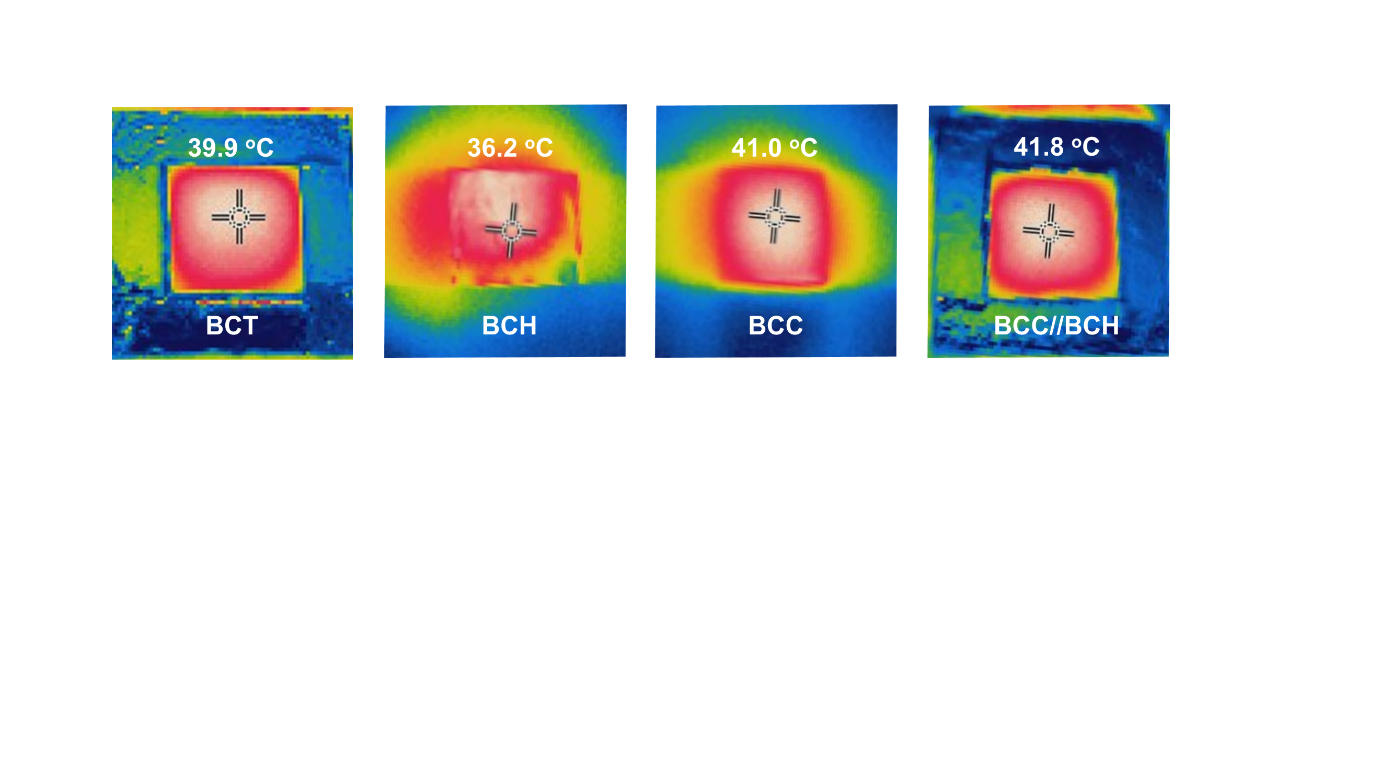


**Fig. S9** Infrared images of the top surfaces of BCT, BCH, BCC, and BCC//BCH membranes, showing their steady-state temperatures under 1-sun irradiation for 60 min


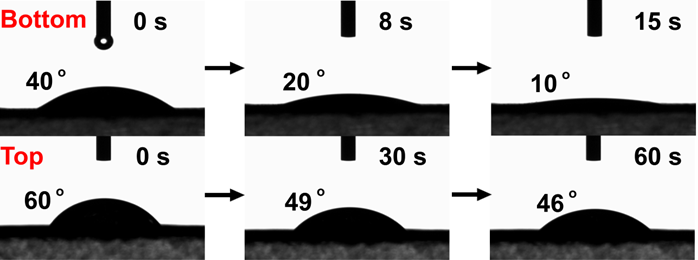


**Fig. S10** Water contact angels of the bottom and the top surfaces of the BCC//BCH membrane


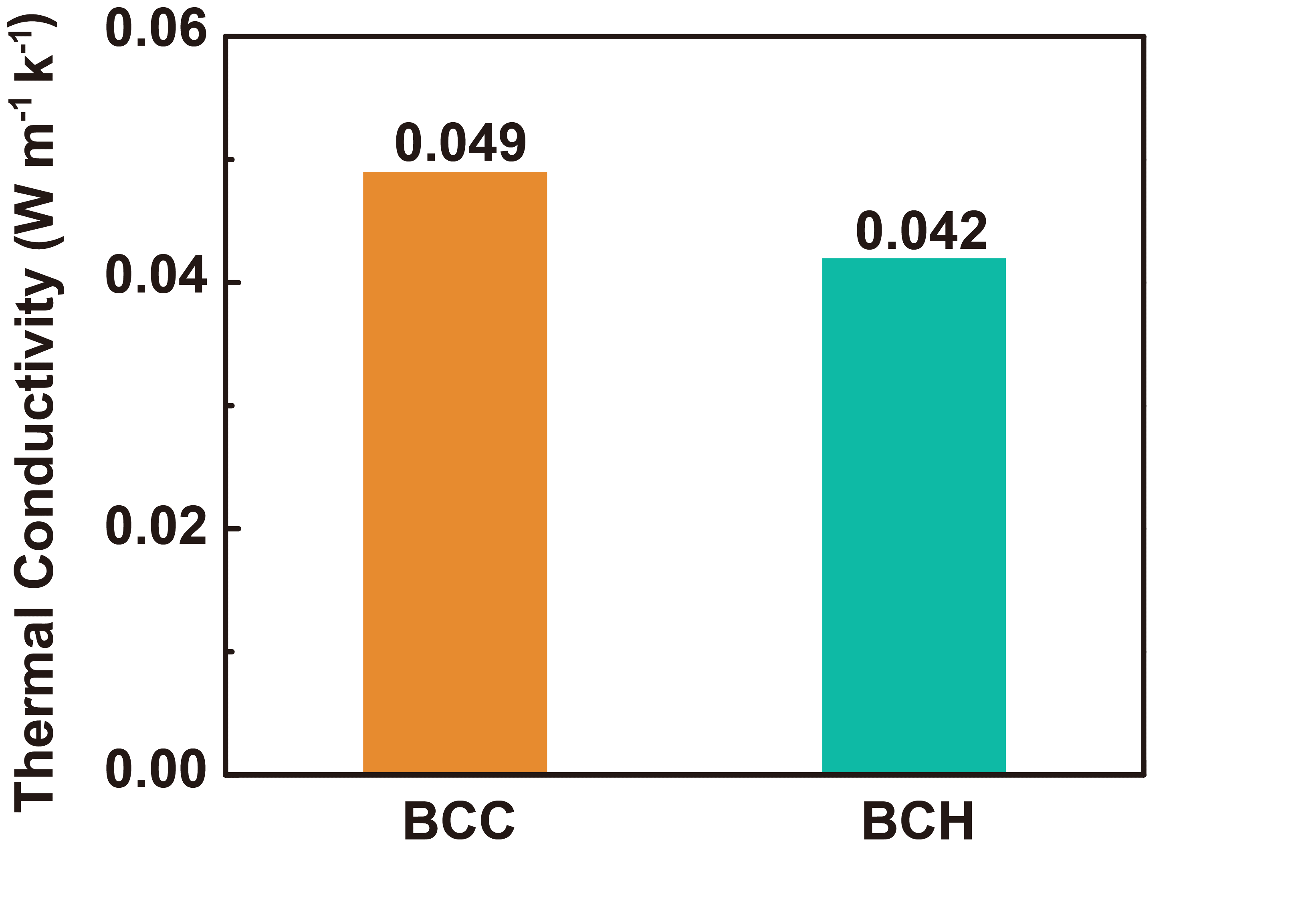


**Fig. S11** Thermal conductivity of BCC and BCH components


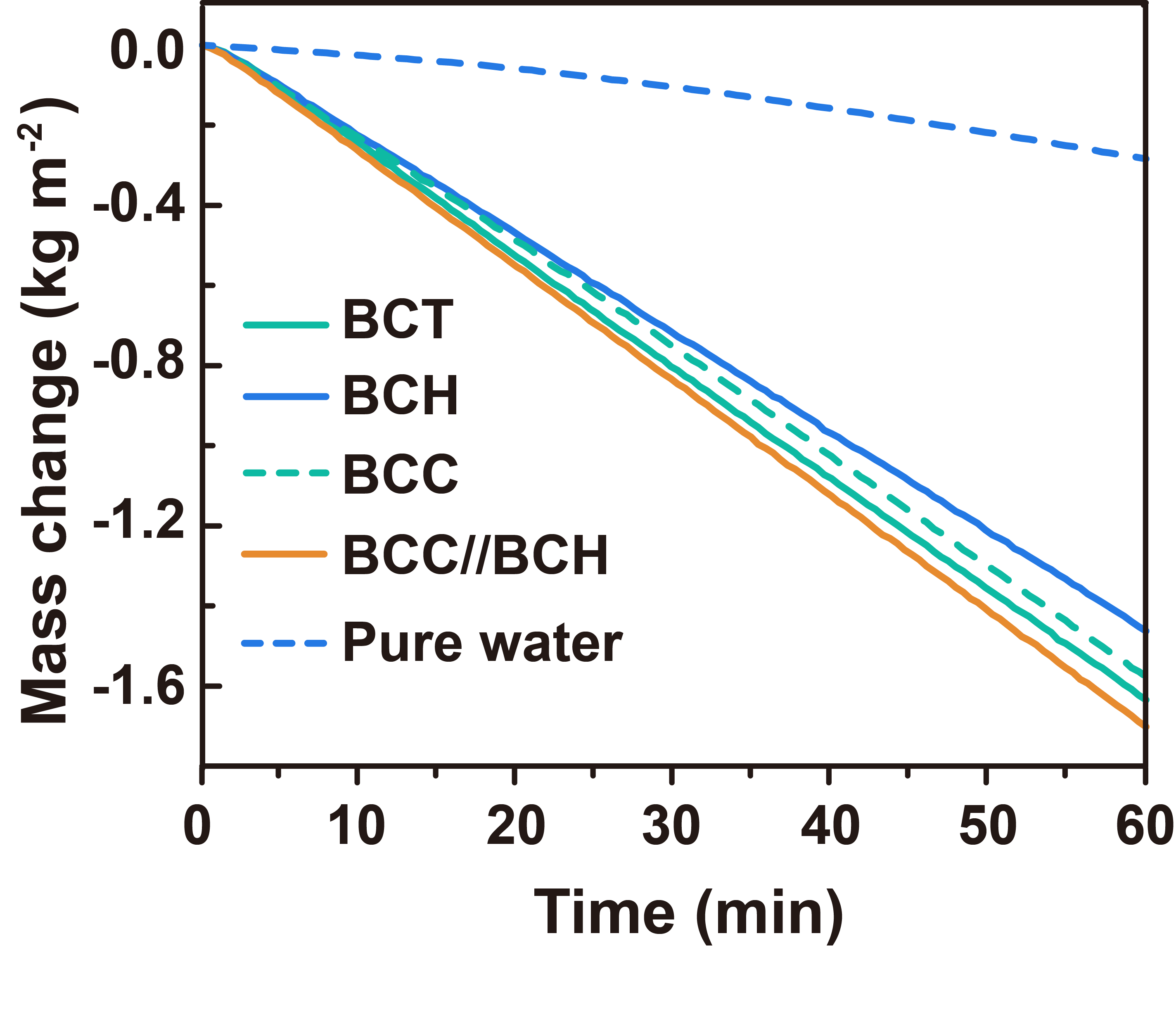


**Fig. S12** Water mass changes of BCT, BCH, BCC and BCC//BCH membranes under 1-sun irradiation


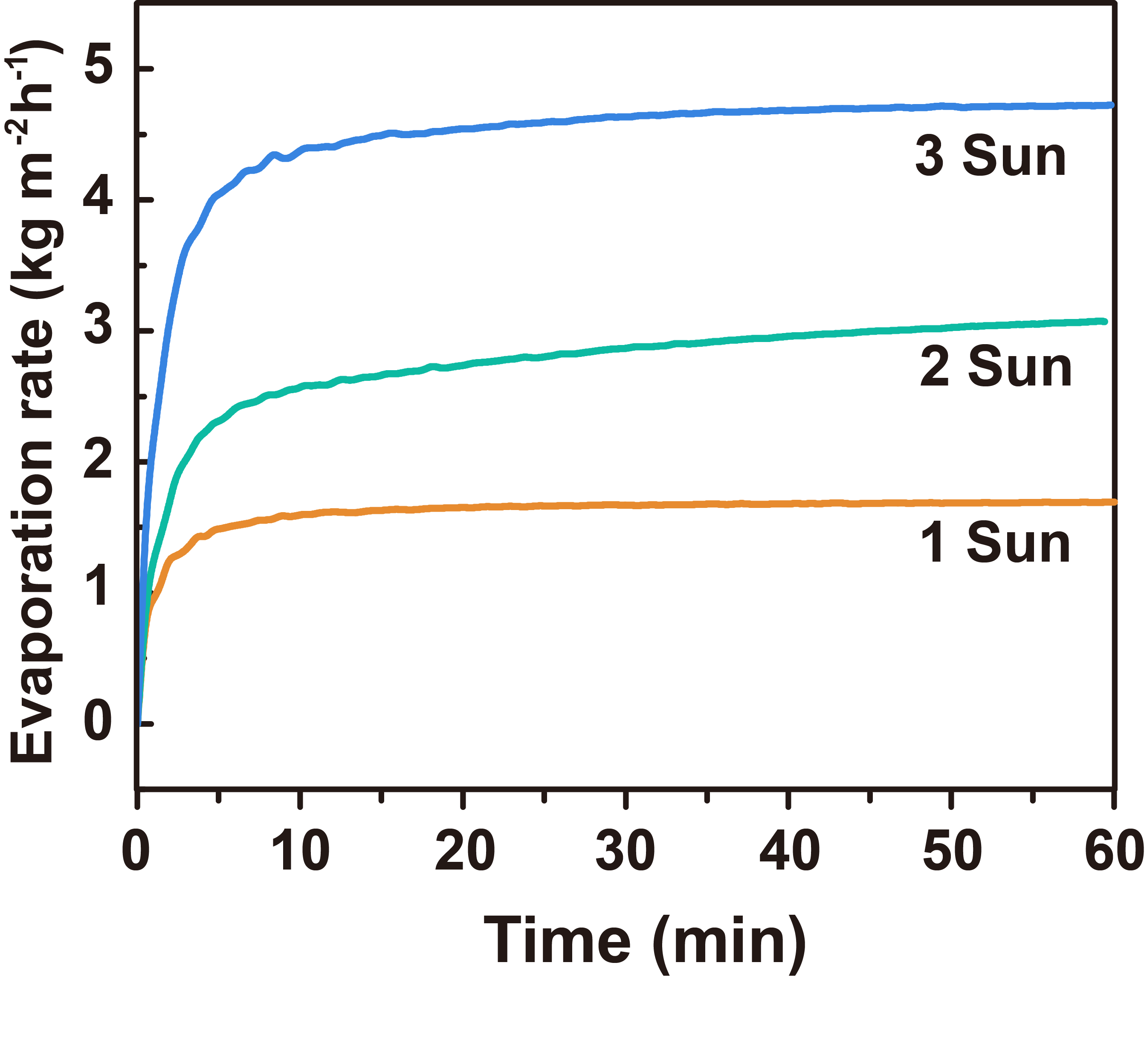


**Fig. S13** Water evaporation rates of the BCC//BCH membrane under different solar irradiatio
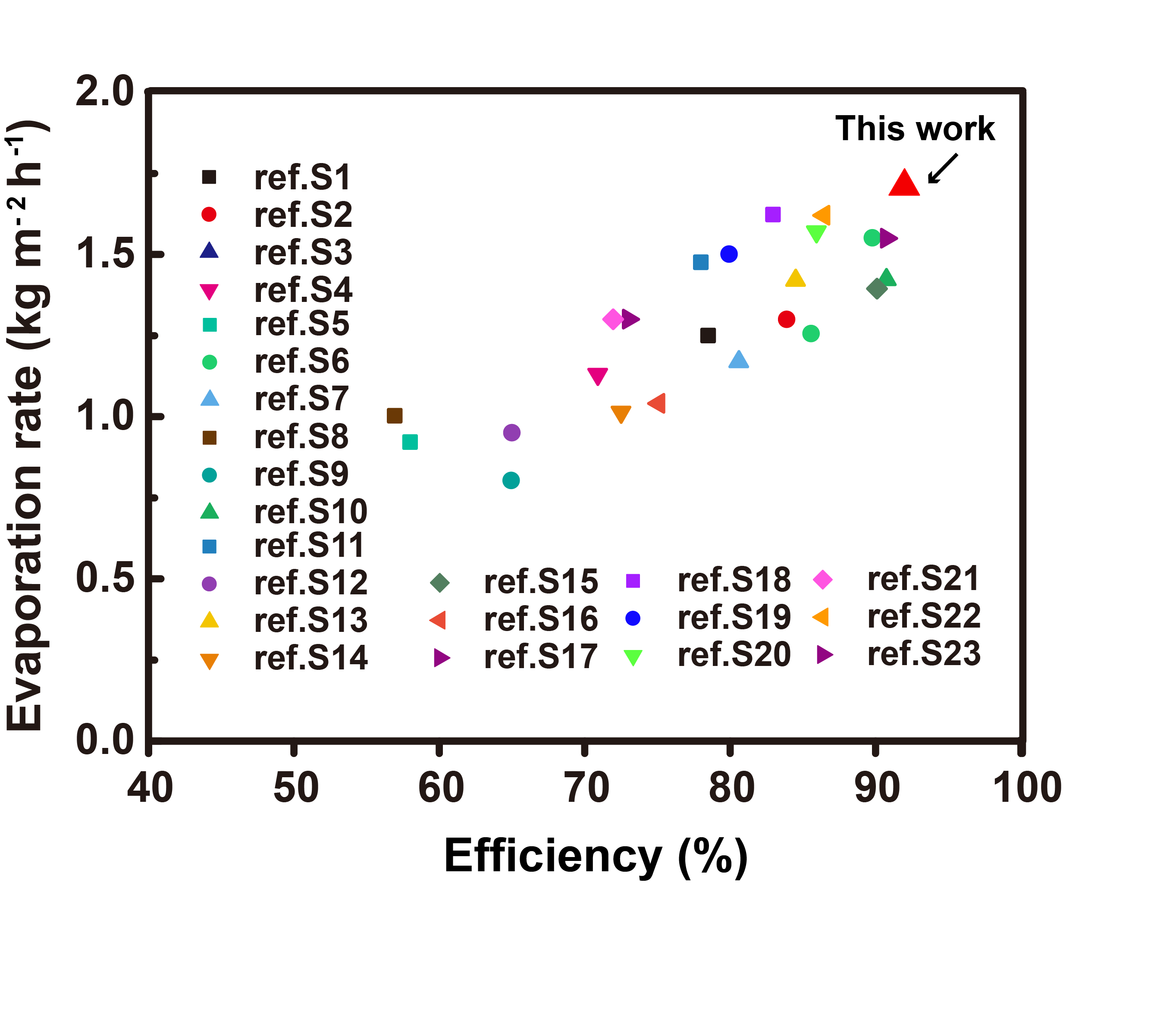


**Fig. S14** Comparison of the evaporation performance of the BCC//BCH bilayer membrane with those of reported solar-driven evaporators [S1-S23]


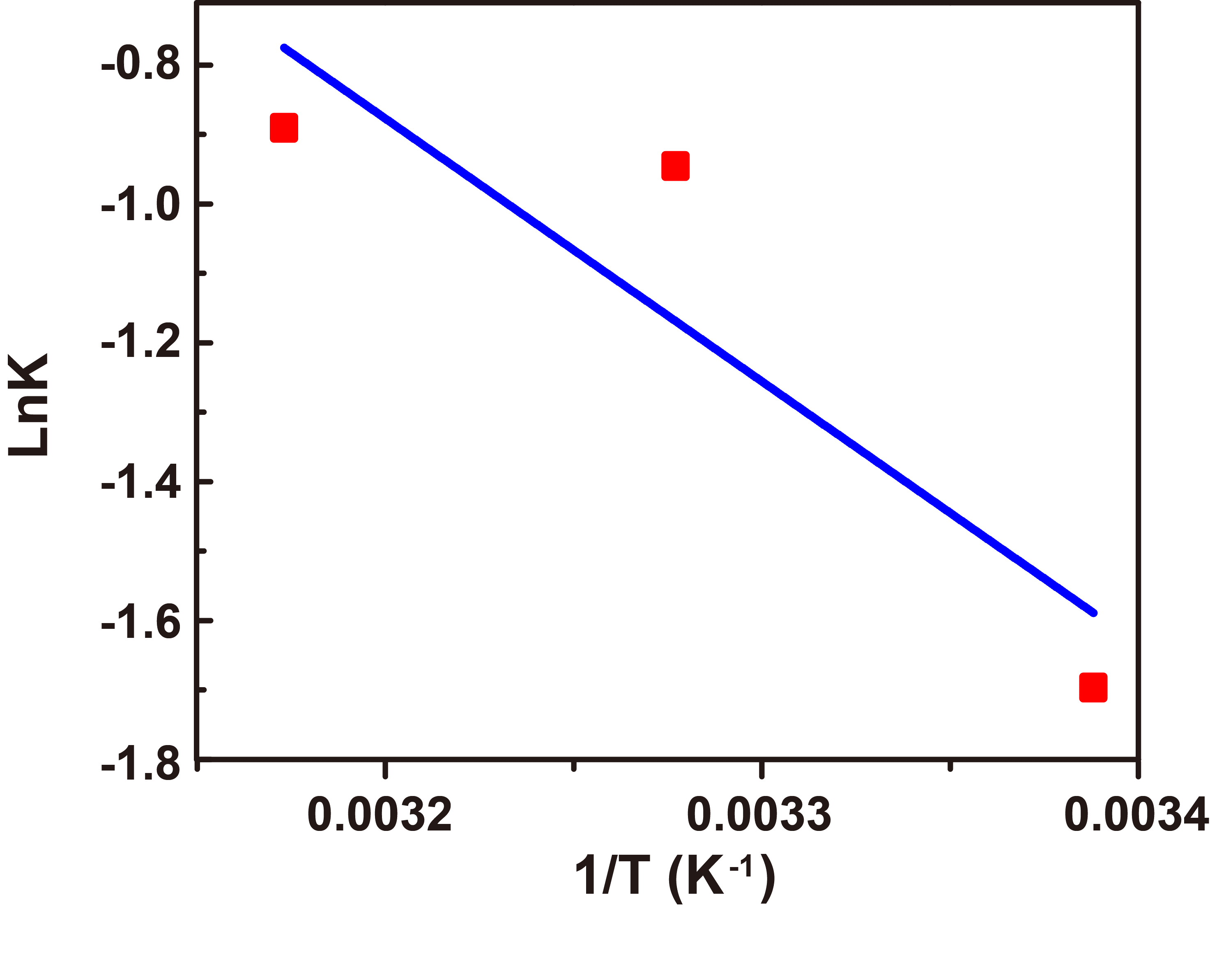


**Fig. S15** Apparent activation energy of the BCC/PMS system


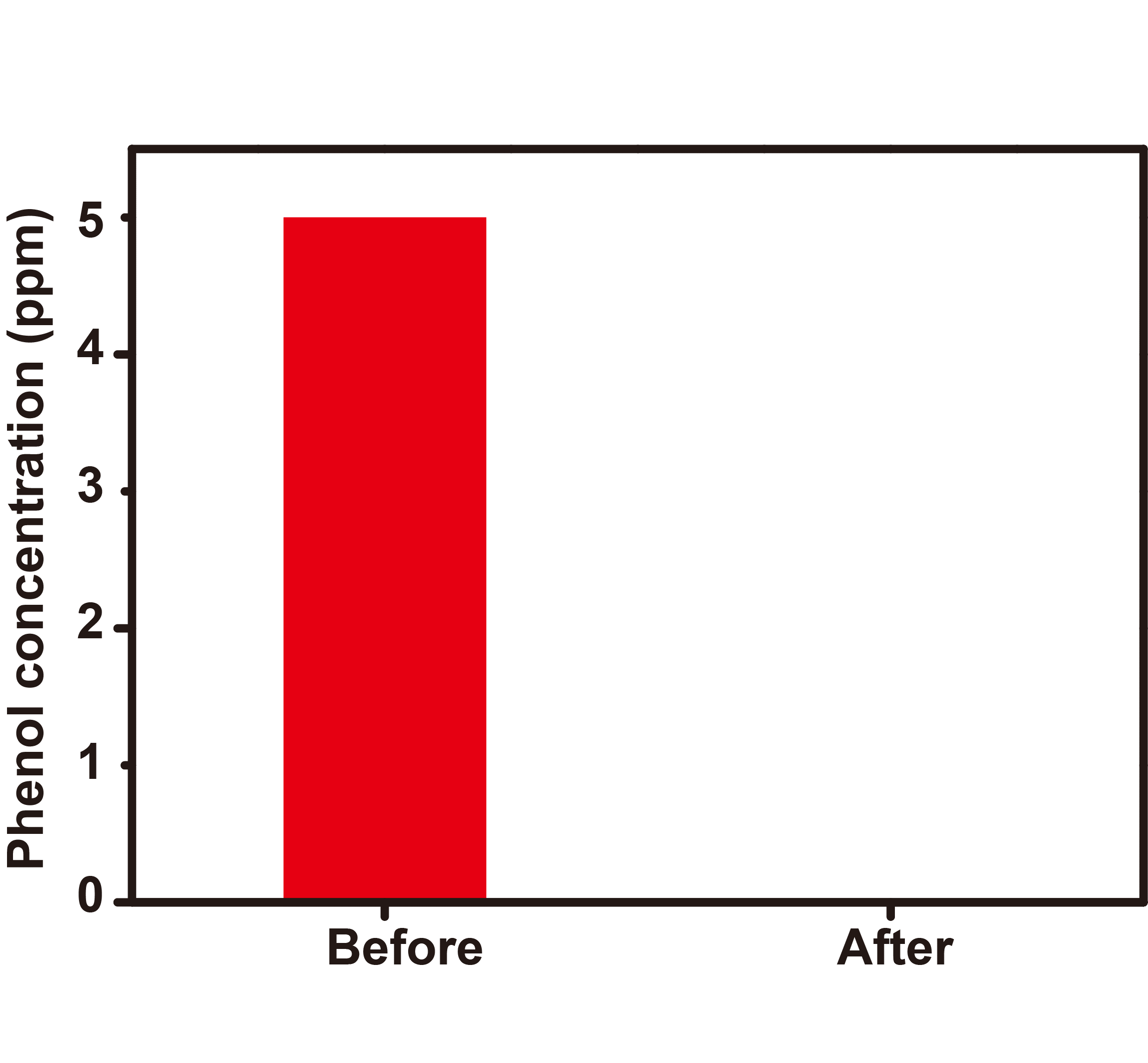


**Fig. S16** Phenol concentration before and after degradation


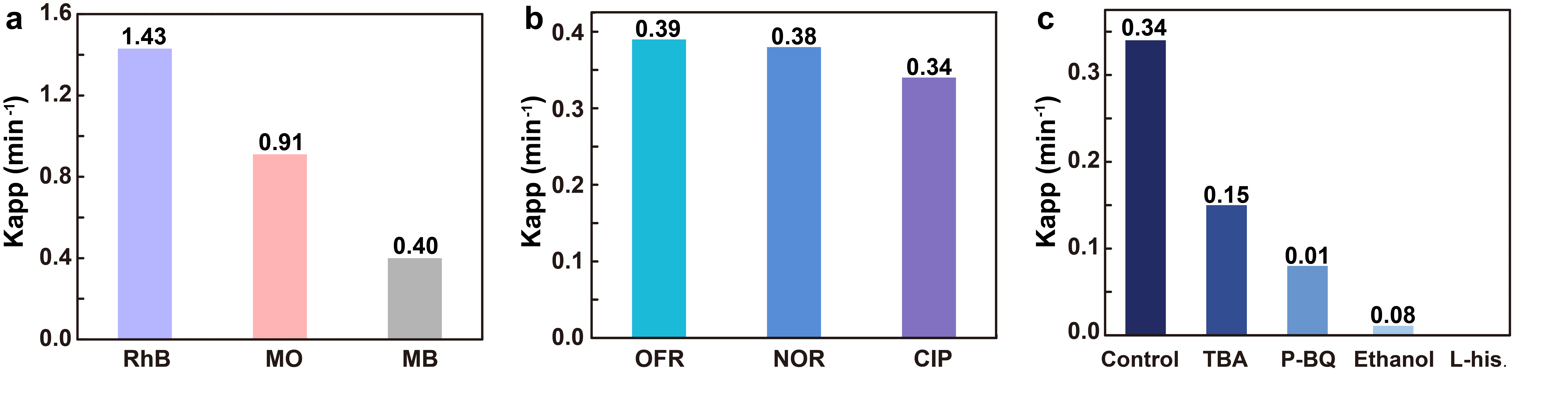


**Fig. S17 a** Degradation rate constant of various dyes using BCC catalyst under 1 kW m^−2^ irradiation. **b** Degradation rate constants of various antibiotics using BCC catalyst under 1 kW m^−2^ irradiation. **c** Degradation rate constants of CIP antibiotics using BCC catalyst under 1 kW m^−2^ irradiation with addition of various scavengers


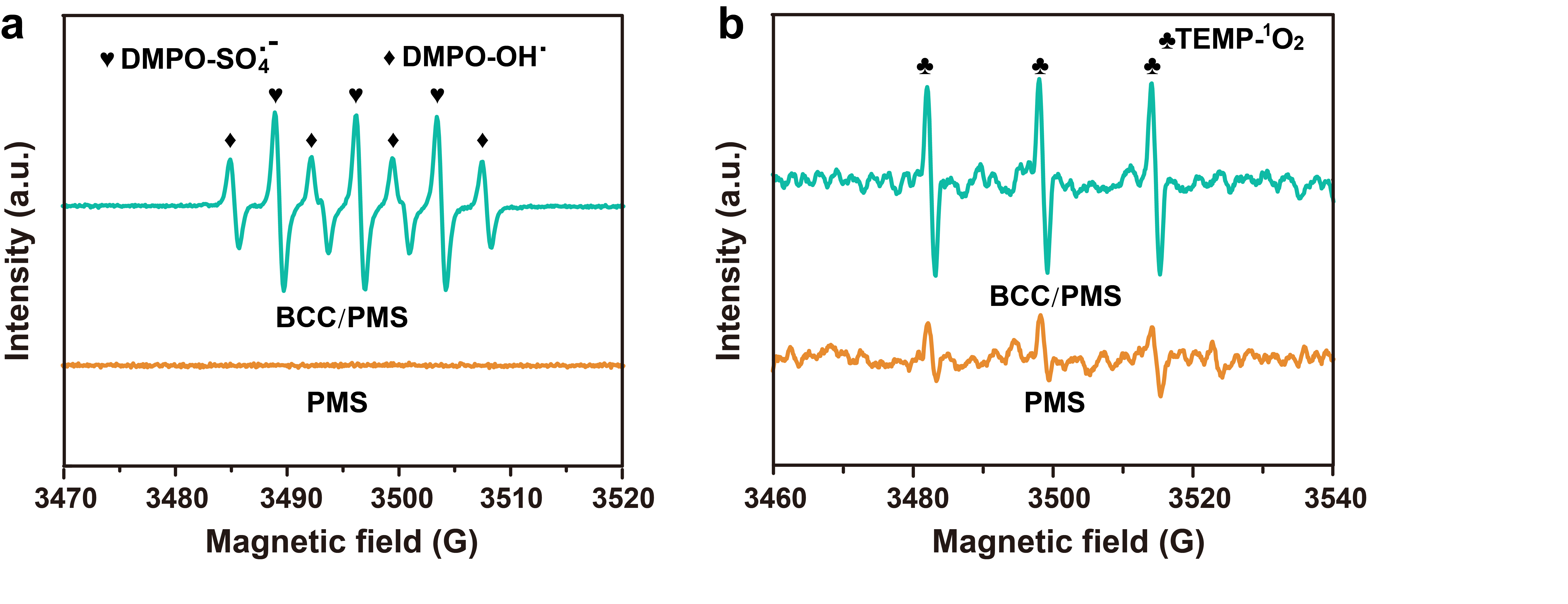


**Fig. S18** EPR spectra of **a** DMPO-^•^OH/SO_4_^•−^ and **b** TEMP-^1^O_2_


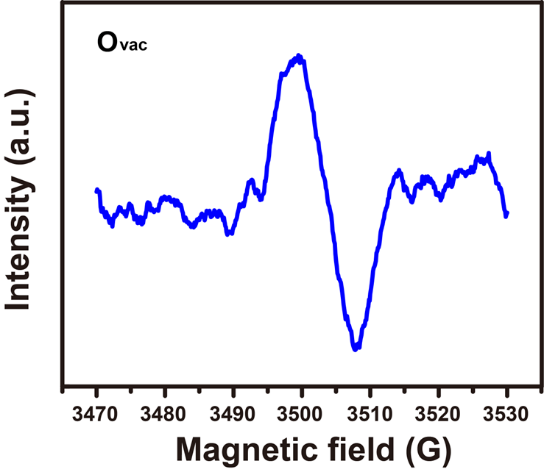


**Fig. S19** EPR spectrum of BCC


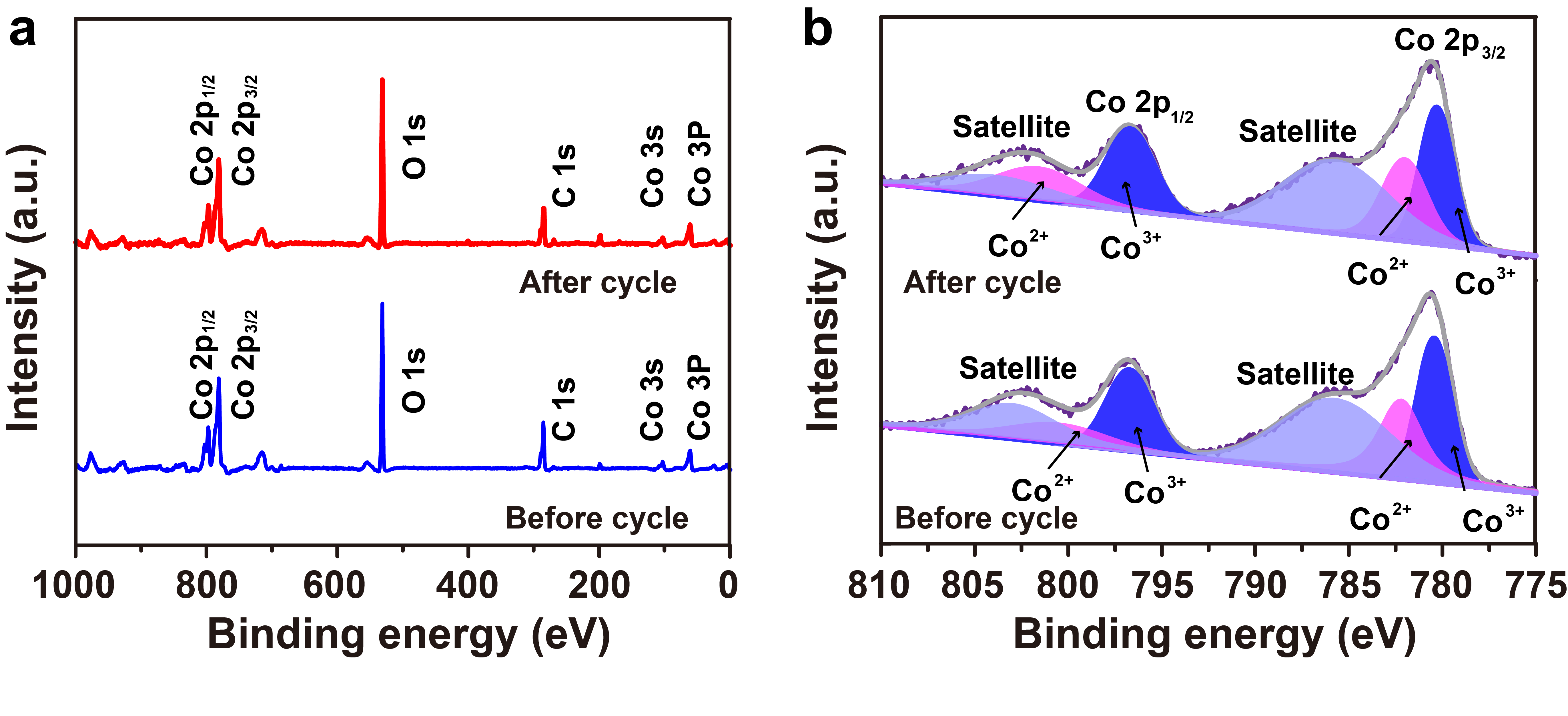


**Fig. S20** **a** XPS and **b** Co 2p spectra before and after the CIP degradation in the BCC/PMS system


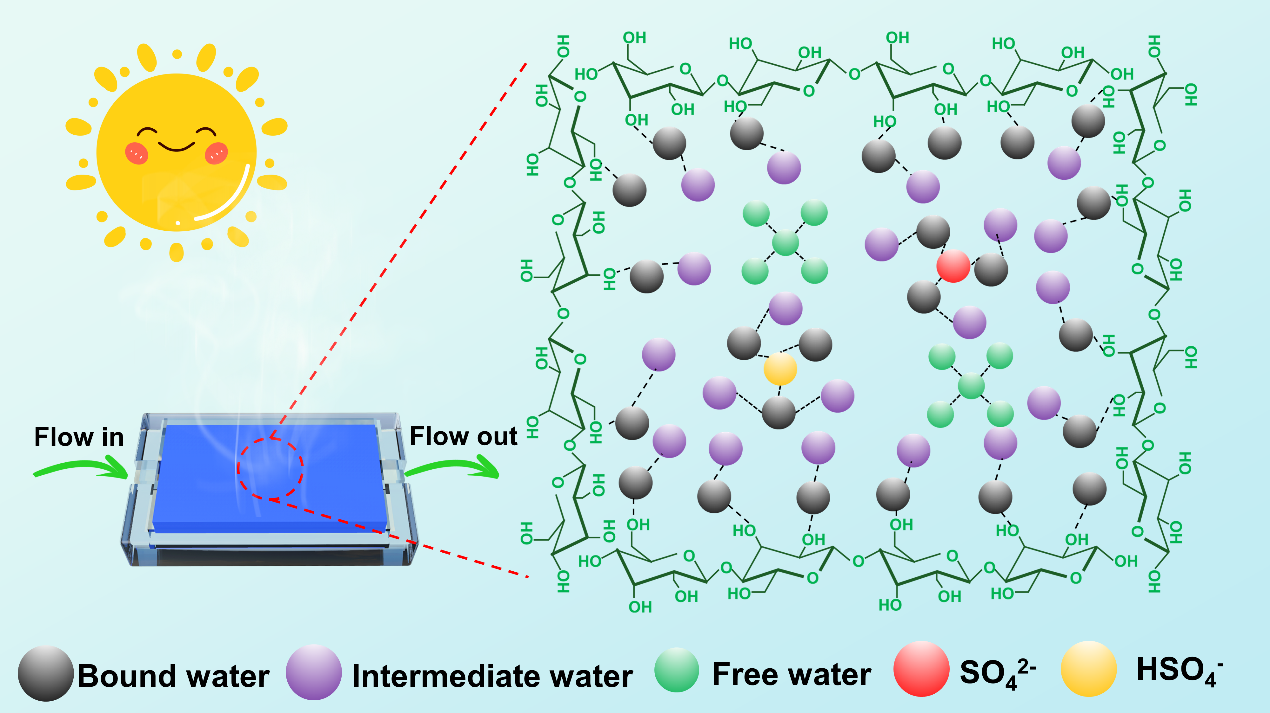


**Fig. S21** The presence of different kinds of water (IW, FW, and BW) in the evaporator


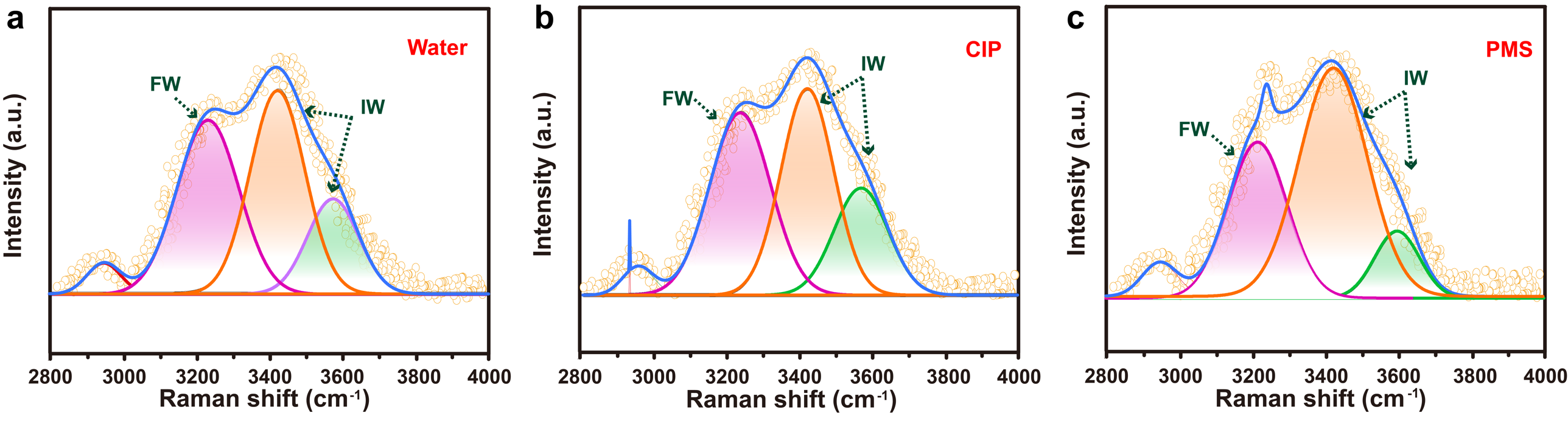


**Fig. S22** Fitting curves based on the Gaussian function in the energy region of the O–H stretching modes of water in **a** BCC//BCH system, **b** BCC//BCH-CIP system, and **c** BCC//BCH-PMS system


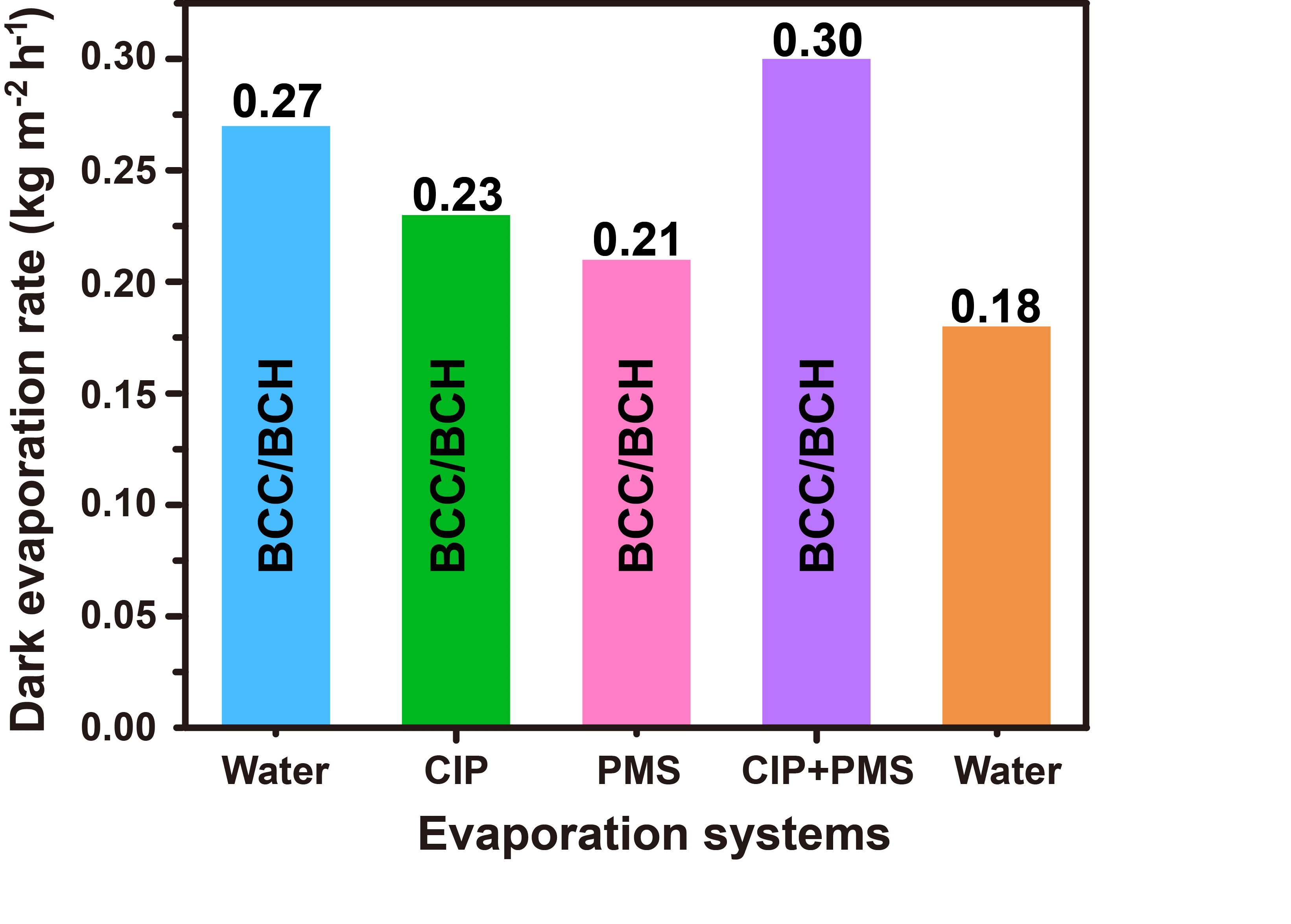


**Fig. S23** Dark evaporation rates of water in different evaporation systems

**
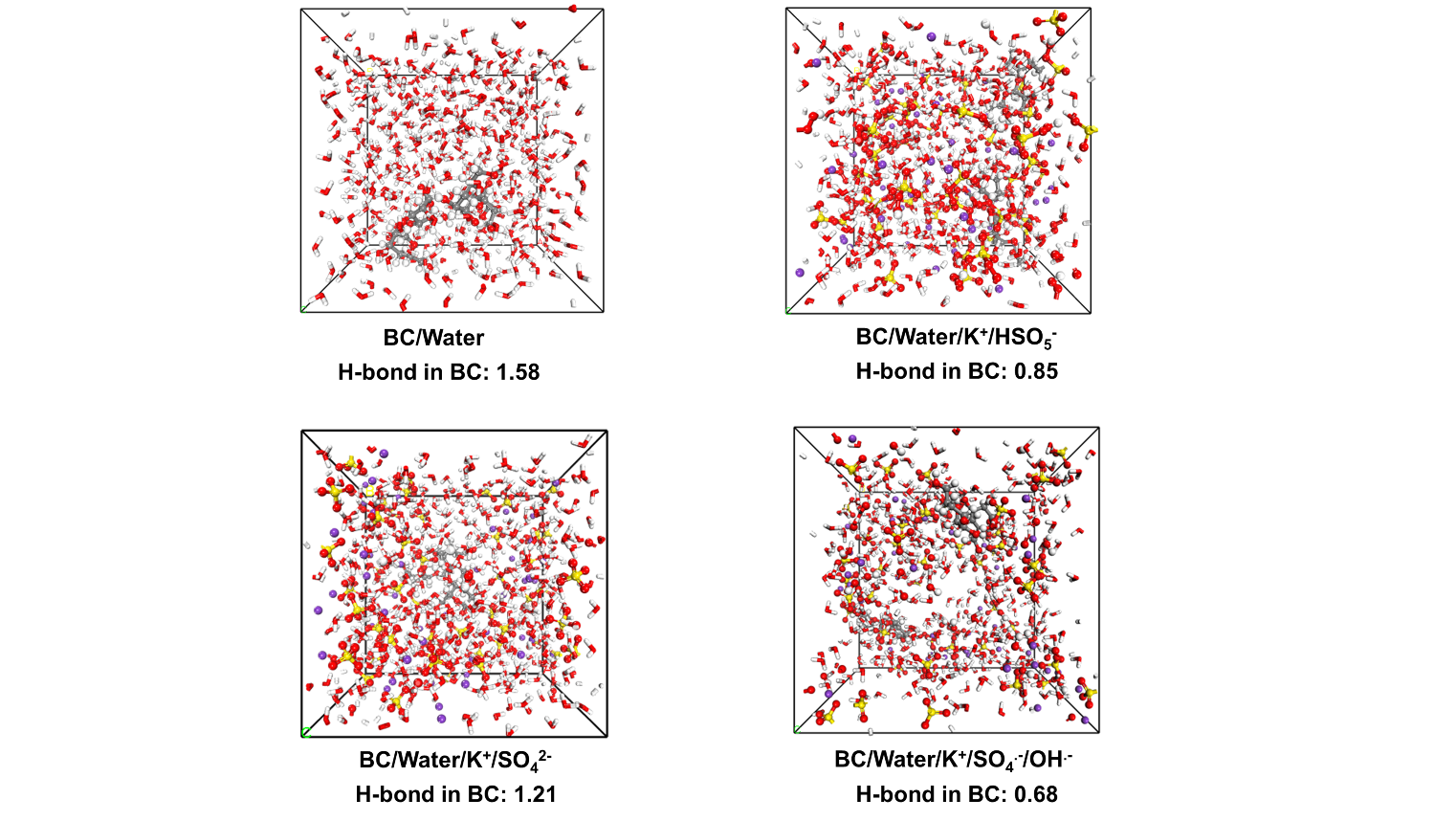
**

**Fig. S24** The average numbers of hydrogen bonds in BC of different systems


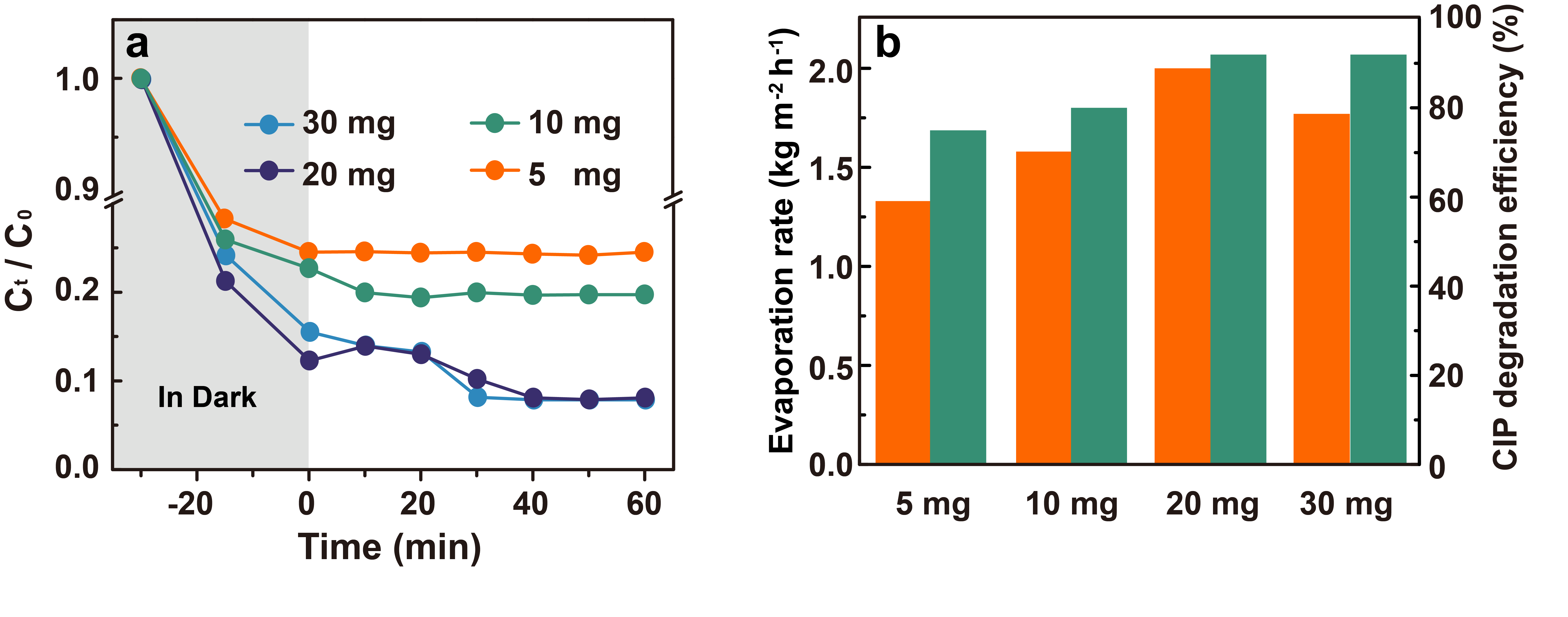


**Fig. S25 a, b** CIP degradation evolution and evaporation rates with different masses of BCC//BCH films


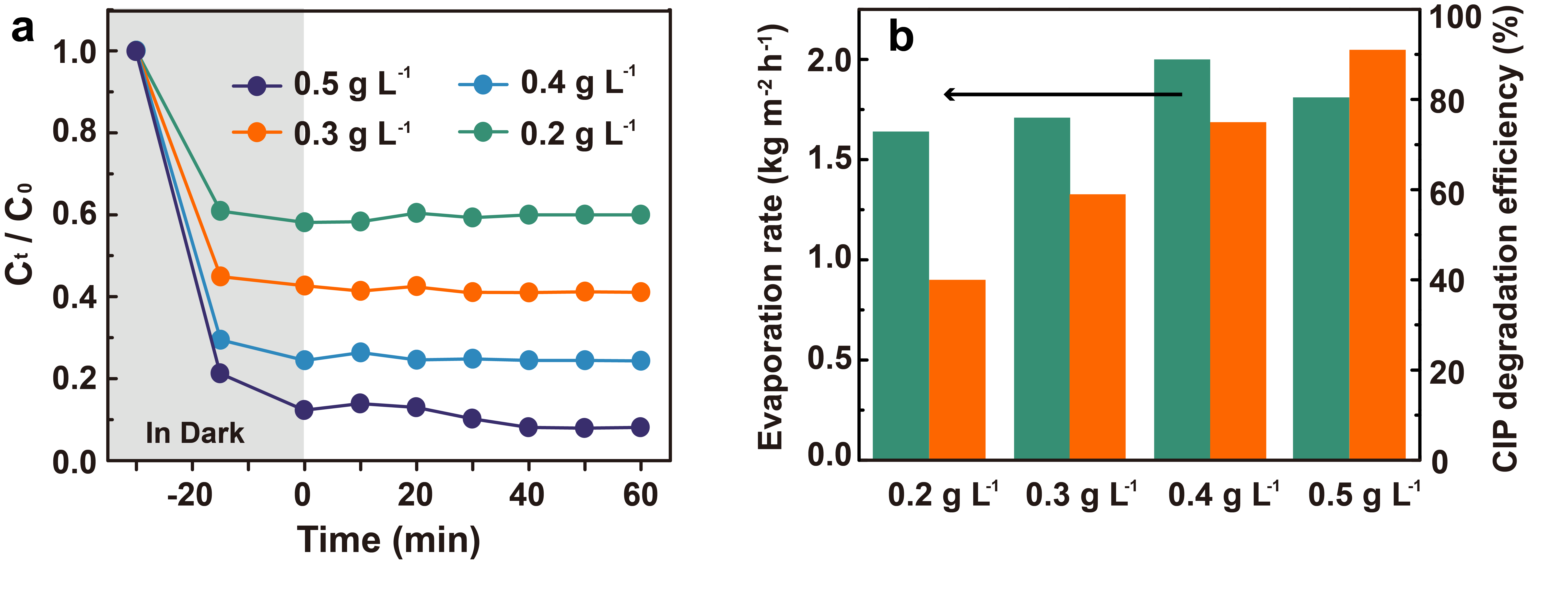


**Fig. S26.** **a, b** CIP degradation evolution and evaporation rates of different PMS concentrations with the optimum BCC//BCH mass


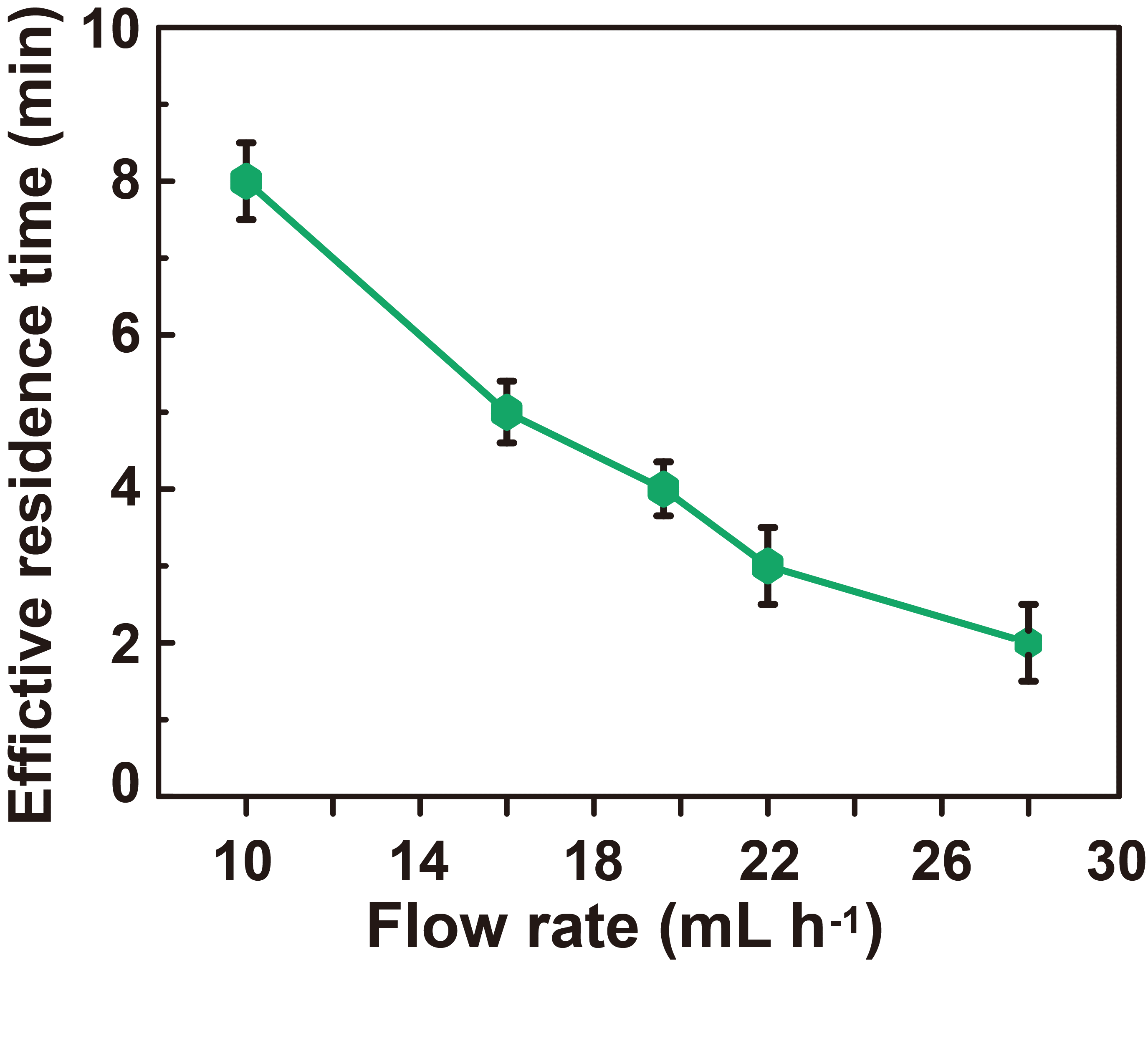


**Fig. S27** Residence times of the CIP pollutant at various flow rates

**
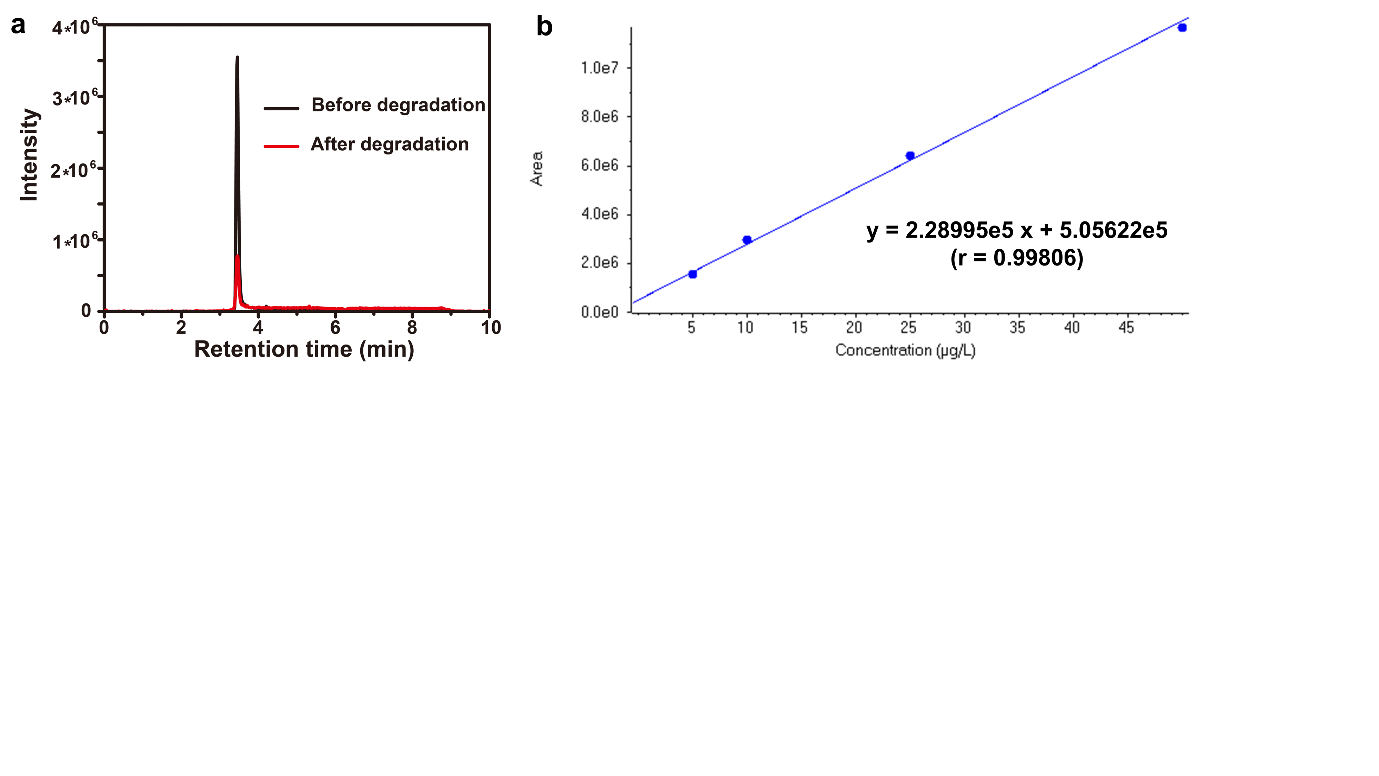
**

**Fig. 28** Concentration of CIP before and after degradation**
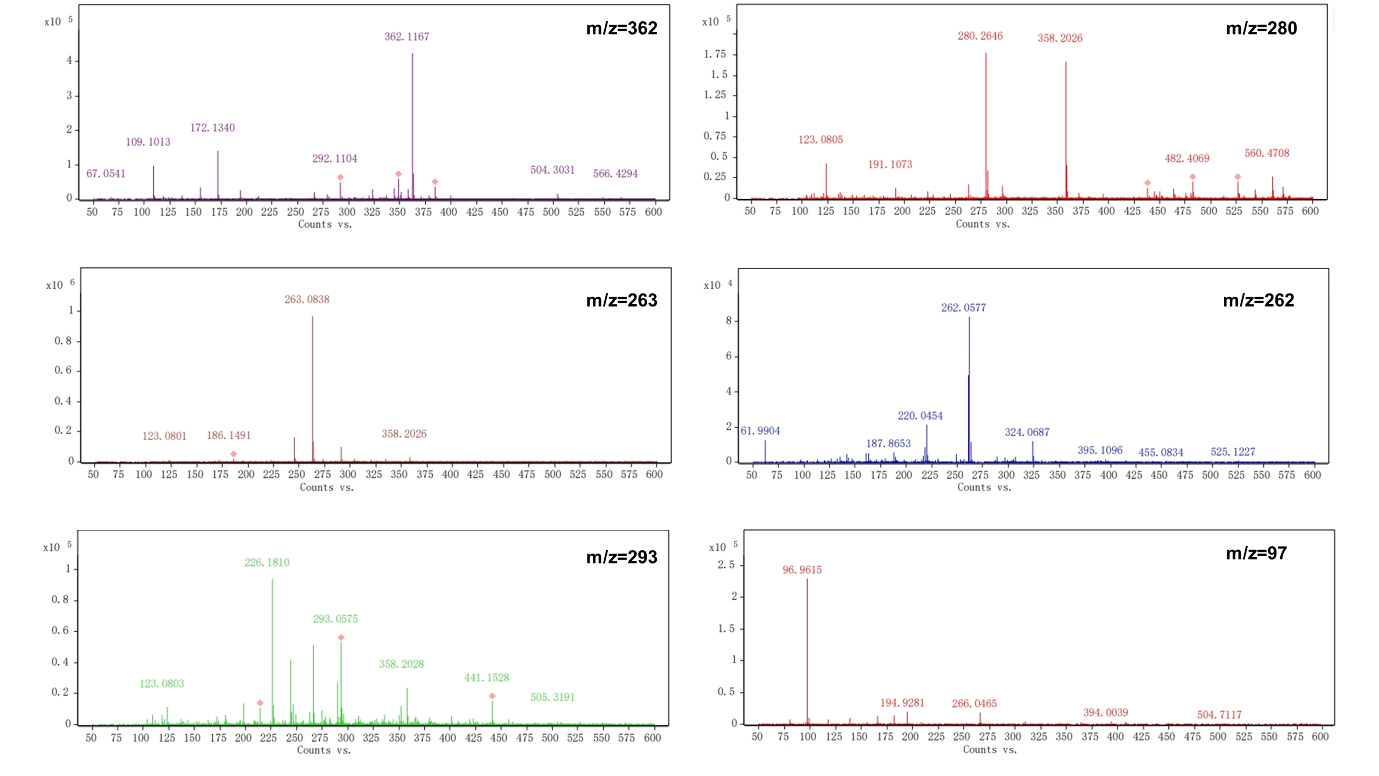
**

**Fig. S29** Mass spectrum of CIP degradation intermediates**
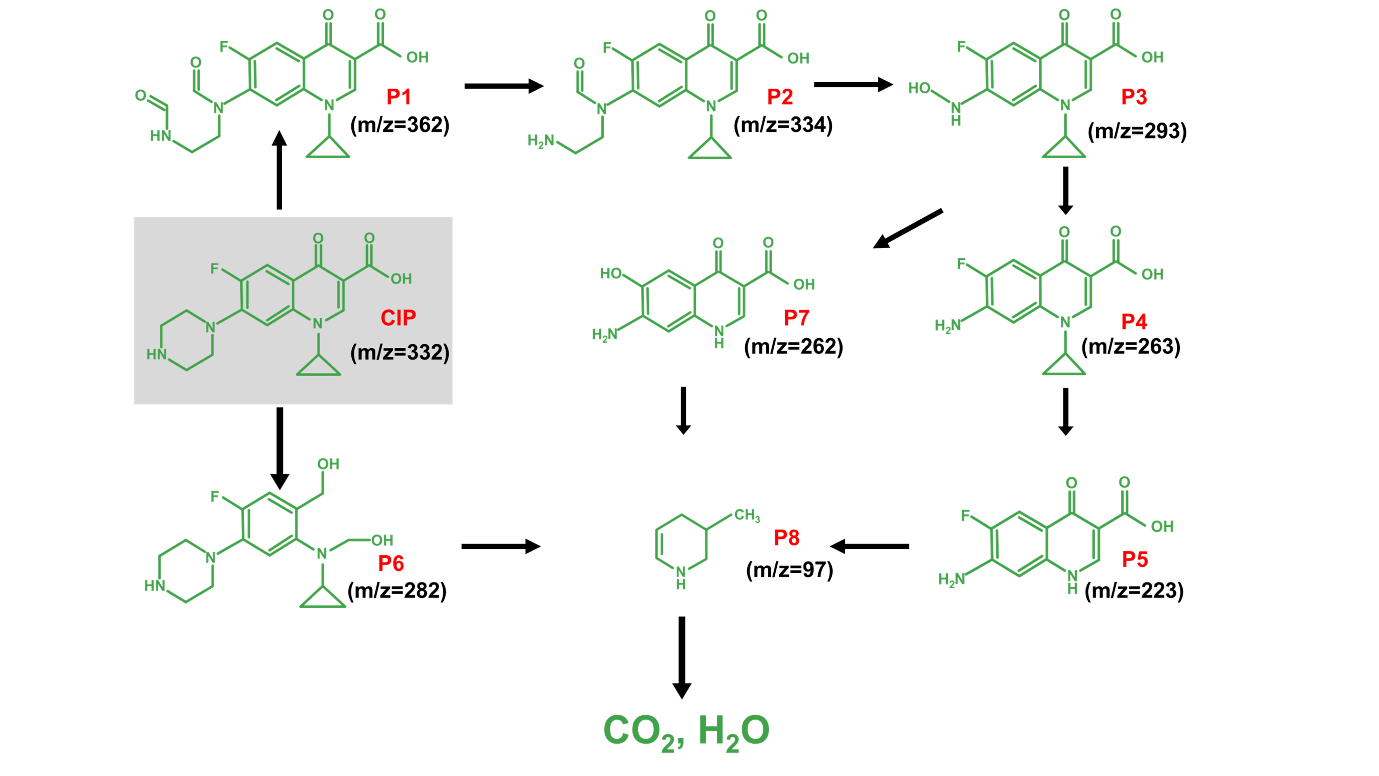
**

**Fig. S30** Proposed degradation pathway of CIP by the flow bed system

**
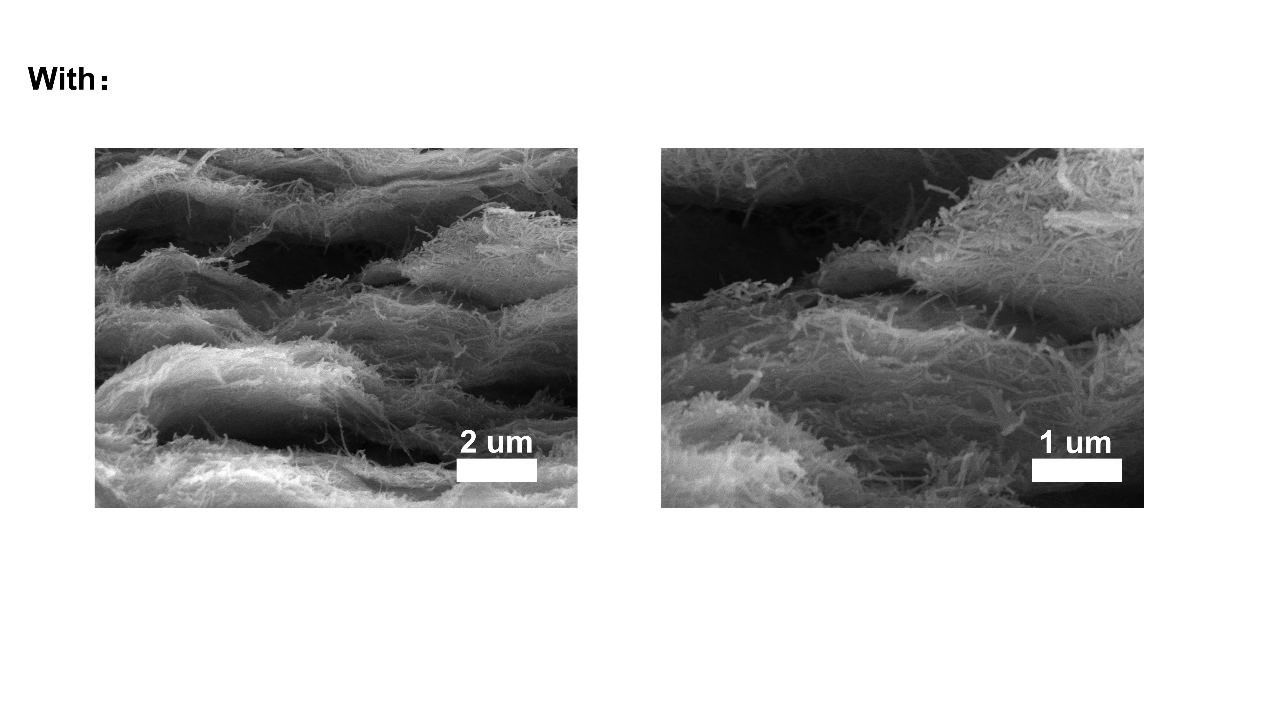
**

**Fig. S31** SEM images of the BCC//BCH interlayers with AOPs

**
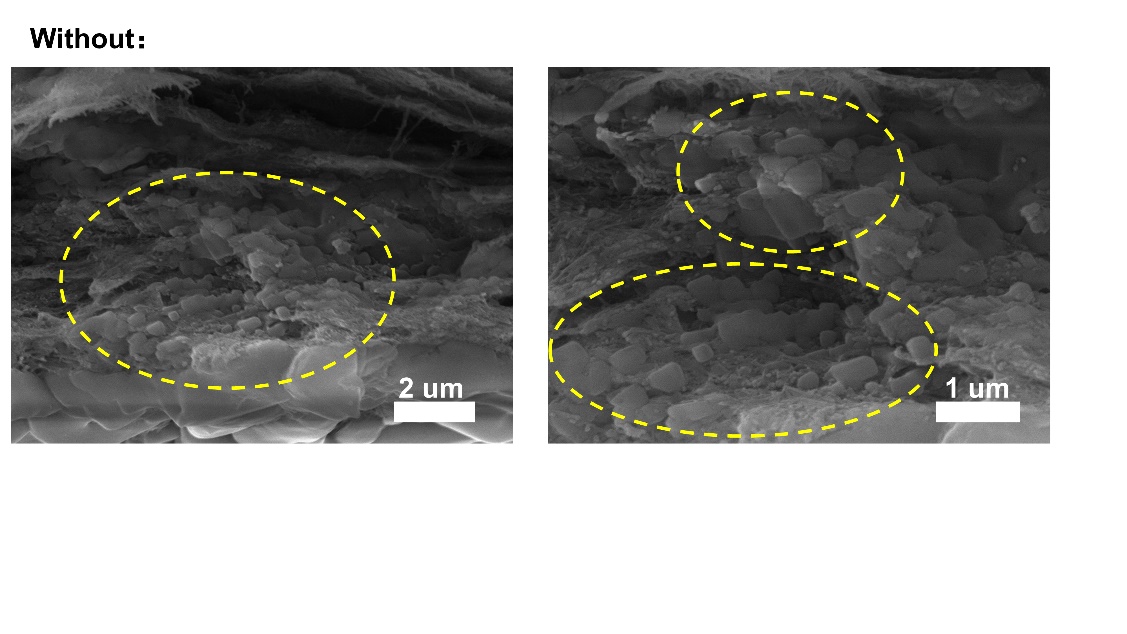
**

**Fig. S32** SEM images of the BCC//BCH interlayers without AOPs


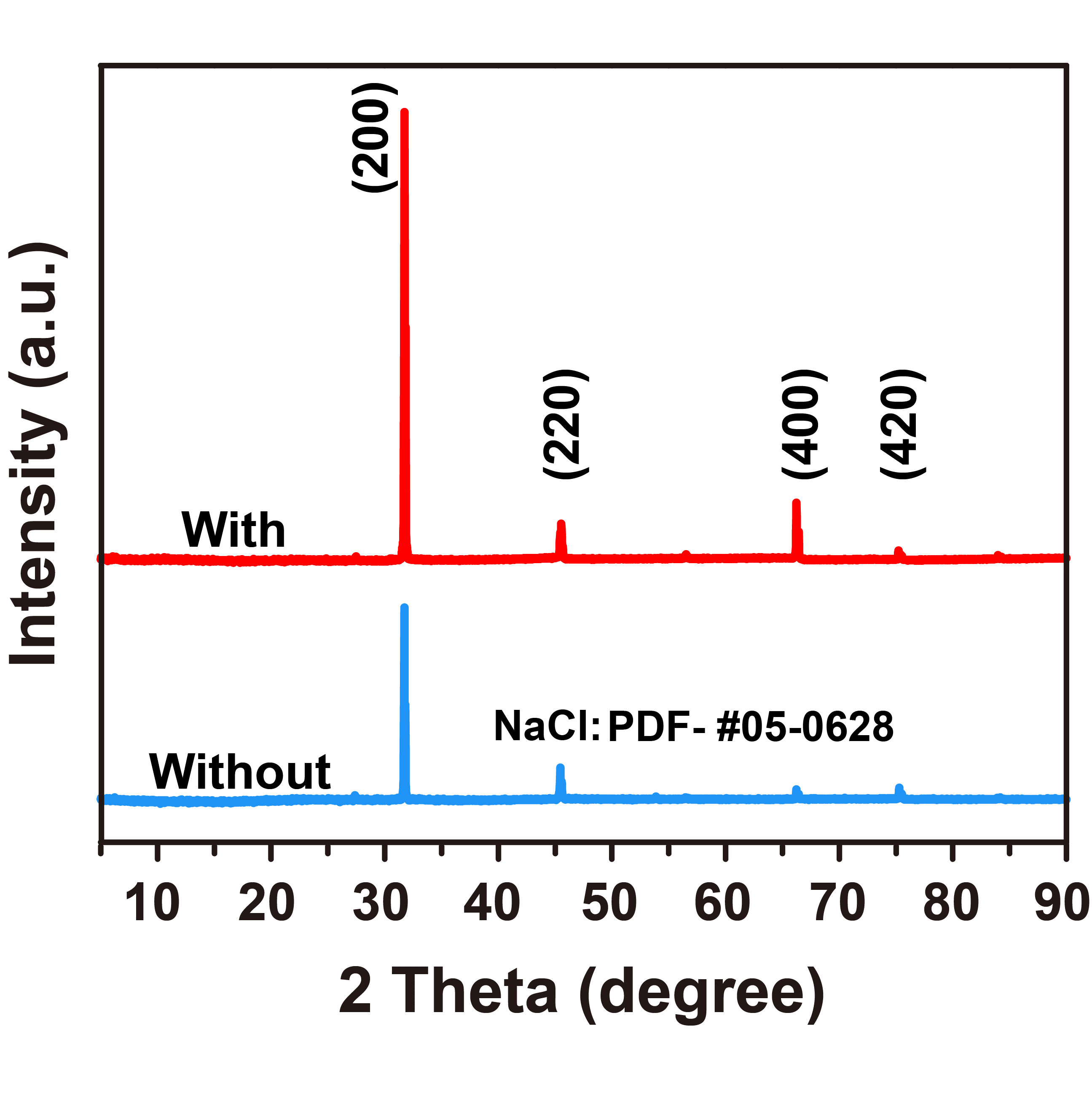


**Fig. S33** XRD patterns of NaCl crystallites formed where PS foam and the BCC//BCH film come into contact during the evaporation processes in 25 wt.% saline

The grain sizes of the NaCl crystals were calculated using the Debye-Scherrer equation:

$$D=\frac{K\lambda}{\beta cos\theta}$$

Where $D$ refers to the grain size (nm), $K$ is the Scherrer constant (0.89), the $\lambda$is the X-ray wavelength and has a value of 0.15406 nm for Cu Ka, $\beta$ is the half peak width of the XRD pattern and θ is the diffraction angel.

**
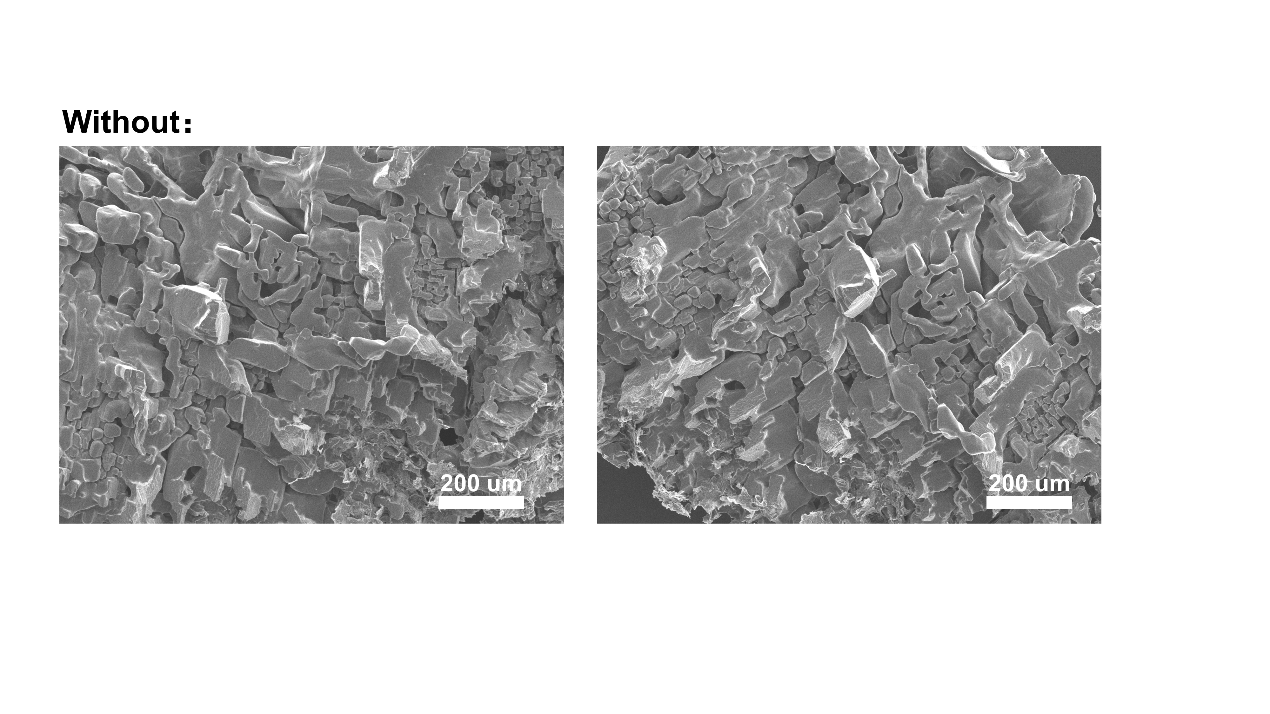
**

**Fig. S34** SEM images of NaCl crystallization at the edge of the BCC//BCH membrane without AOP_S_

**
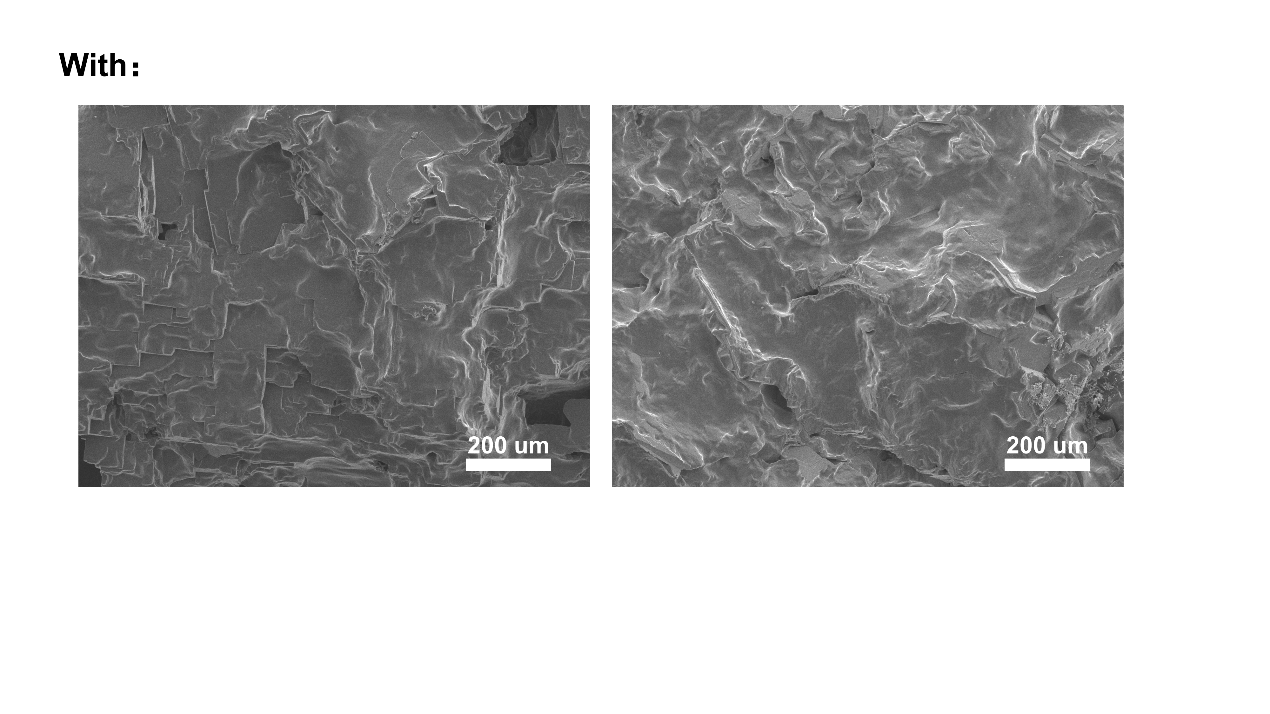
**

**Fig. S35** SEM images of NaCl crystallization at the edge of the BCC//BCH membrane with AOP_S_

**Table S1** Comparison of the solar steam generation performances of the BCC//BCH evaporator with those of other solar-thermal materials under 1-sun irradiation

| Entry | Materials | Evaporation rate  (kg m^-2^ h^-1^) | Efficiency  (%) | References |
| --- | --- | --- | --- | --- |
| 1 | **BCC//BCH** | **1.70** | **93%** | **This work** |
| 2 | LAL | 1.25 | 78.5 | [S1] |
| 3 | MoOx HNS Membrane | 1.26 | 85.6 | [S2] |
| 4 | TiOx | 0.80 | 50.3 | [S3] |
| 5 | H-TiO_2_ | 1.13 | 70.9 | [S4] |
| 6 | Ppy-coated mesh | 0.92 | 58.0 | [S5] |
| 7 | Oligoaniline | 1.17 | 80.6 | [S6] |
| 8 | PIP2 | 1.55 | 89.8 | [S7] |
| 9 | AI NP/AAM | 1.00 | 57.0 | [S8] |
| 10 | AU/D-NPT | 0.80 | 65.0 | [S9] |
| 11 | Aluminophosphate-treated wood | 1.42 | 90.8 | [S10] |
| 12 | Mushroom | 1.48 | 78.0 | [S11] |
| 13 | F-Wood/CNTs | 0.95 | 65.0 | [S12] |
| 14 | C800-ZIF-8 WS | 1.42 | 84.5 | [S13] |
| 15 | PPy–wood | 1.01 | 72.5 | [S14] |
| 16 | AGW | 1.39 | 90.1 | [S15] |
| 17 | Self-regenerating evaporator | 1.04 | 85.1 | [S16] |
| 18 | Fe-D-Wood | 1.30 | 73.0 | [S17] |
| 19 | RGO-SA-CNT aerogels | 1.62 | 83.0 | [S18] |
| 20 | N950 porous graphene | 1.50 | 80.0 | [S19] |
| 21 | SPCF | 1.57 | 86.0 | [S20] |
| 22 | CB/PMMA-PAN | 1.30 | 72.0 | [S21] |
| 23 | VA-GSM | 1.62 | 86.5 | [S22] |
| 24 | PSN-rGO aerogel | 1.55 | 90.8 | [S23] |

**Supplementary References**

1. X. Chen, C. Meng, Y. Wang, Q. Zhao, Y. Li et al., Laser-synthesized rutile TiO_2_ with abundant oxygen vacancies for enhanced solar water evaporation. ACS Sustainable Chem. Eng. **8**(2), 1095-1101 (2020). <https://doi.org/10.1021/acssuschemeng.9b05952>
2. Q. Lu, Y. Yang, J. Feng, X. Wang, Oxygen‐defected molybdenum oxides hierarchical nanostructure constructed by atomic‐level thickness nanosheets as an efficient absorber for solar steam generation. Solar RRL **3**(2), 1800277 (2018). <https://doi.org/10.1002/solr.201800277>
3. M. Ye, J. Jia, Z. Wu, C. Qian, R. Chen et al., Synthesis of black TiO_x_ nanoparticles by Mg reduction of TiO_2_ nanocrystals and their application for solar water evaporation. Adv. Energy Mater. **7**(4), 1601811 (2016). <https://doi.org/10.1002/aenm.201601811>
4. G. Zhu, J. Xu, W. Zhao, F. Huang. Constructing Black Titania with Unique Nanocage Structure for Solar Desalination. ACS Appl. Mater. Interfaces **8**(46), 31716-31721 (2016). <https://doi.org/10.1021/acsami.6b11466>
5. L. Zhang, B. Tang, J. Wu, R. Li, P. Wang. Hydrophobic light‐to‐heat conversion membranes with self‐healing ability for interfacial solar heating. Adv. Mater. **27**(33), 4889-4894 (2015). <https://doi.org/10.1002/adma.201502362>
6. Q. Chen, Z. Pei, Y. Xu, Z. Li, Y. Yang et al., A Durable Monolithic Polymer Foam for Efficient Solar Steam Generation. Chem. Sci. **9**(3), 623-628 (2018). <https://doi.org/10.1039/c7sc02967e>
7. F. Wang, Y. Su, Y. Li, D. Wei, H. Sun et al., Salt-resistant photothermal materials based on monolithic porous ionic polymers for efficient solar steam generation. ACS Appl. Energy Mater. **3**(9), 8746-8754 (2020). <https://doi.org/10.1021/acsaem.0c01292>
8. L. Zhou, Y. Tan, J. Wang, W. Xu, Y. Yuan et al., 3D Self-assembly of aluminium nanoparticles for plasmon-enhanced solar desalination. Nat. Photonics **10**(6), 393-398 (2016). <https://doi.org/10.1038/nphoton.2016.75>
9. L. Zhou, Y. Tan, D. Ji, B. Zhu, P. Zhang et al., Self-assembly of highly efficient, broadband plasmonic absorbers for solar steam generation. Sci. Adv. **2**(4), e1501227 (2016). <https://doi.org/10.1126/sciadv.1501227>
10. T. Chen, Z. Wu, Z. Liu, J.T. Aladejana, X. Wang et al., Hierarchical Porous Aluminophosphate-Treated Wood for High-Efficiency Solar Steam Generation. ACS Appl. Mater. Interfaces **12**(17), 19511-19518 (2020). <https://doi.org/10.1021/acsami.0c01815>
11. N. Xu, X. Hu, W. Xu, X. Li, L. Zhou et al., Mushrooms as efficient solar steam‐generation devices. Adv. Mater. **29**(28), 1606762 (2017). <https://doi.org/10.1002/adma.201606762>
12. C. Chen, Y. Li, J. Song, Z. Yang, Y. Kuang et al., Highly flexible and efficient solar steam generation device. Adv. Mater. **29**(30), 1701756 (2017). <https://doi.org/10.1002/adma.201701756>
13. T. Meng, Z. Li, Z. Wan, J. Zhang, L. Wang et al., MoF-derived nanoarchitectured carbons in wood sponge enable solar-driven pumping for high-efficiency soil water extraction. Chem. Eng. J. **452**, 139193 (2023). <https://doi.org/10.1016/j.cej.2022.139193>
14. Z. Wang, Y. Yan, X. Shen, C. Jin, Q. Sun et al., A Wood–polypyrrole composite as a photothermal conversion device for solar evaporation enhancement. J. Mater. Chem. A **7**(36), 20706-20712 (2019). <https://doi.org/10.1039/c9ta04914b>
15. Q. Zhang, L. Li, B. Jiang, H. Zhang, N. He et al., Flexible and mildew-resistant wood-derived aerogel for stable and efficient solar desalination. ACS Appl. Mater. Interfaces **12**(25), 28179-28187 (2020). <https://doi.org/10.1021/acsami.0c05806>
16. Y. Kuang, C. Chen, S. He, E. M. Hitz, Y. Wang, W. Gan, R. Mi, L. Hu. A high‐performance self‐regenerating solar evaporator for continuous water desalination. Adv. Mater. **31**(23), 1900498 (2019). <https://doi.org/10.1002/adma.201900498>
17. L. Song, X.-F. Zhang, Z. Wang, T. Zheng, J. Yao. Fe_3_O_4_/polyvinyl alcohol decorated delignified wood evaporator for continuous solar steam generation. Desalination **507**, 115024 (2021). <https://doi.org/10.1016/j.desal.2021.115024>
18. X. Hu, W. Xu, L. Zhou, Y. Tan, Y. Wang et al., Tailoring graphene oxide‐based aerogels for efficient solar steam generation under one sun. Adv. Mater. **29**(5), 1604031 (2016). <https://doi.org/10.1002/adma.201604031>
19. Y. Ito, Y. Tanabe, J. Han, T. Fujita, K. Tanigaki et al., Multifunctional porous graphene for high‐efficiency steam generation by heat localization. Adv. Mater. **27**(29), 4302-4307 (2015). <https://doi.org/10.1002/adma.201501832>
20. C. Wang, J. Wang, Z. Li, K. Xu, T. Lei et al., Super-hydrophilic porous carbon foam as a self-desalting monolithic solar steam generation device with high energy efficiency. J. Mater. Chem. A **8**(19), 9528-9535 (2020). <https://doi.org/10.1039/d0ta01439g>
21. W. Xu, X. Hu, S. Zhuang, Y. Wang, X. Li et al., Flexible and salt resistant janus absorbers by electrospinning for stable and efficient solar desalination. Adv. Energy Mater. **8**(14), 1702884 (2018). <https://doi.org/10.1002/aenm.201702884>
22. P. Zhang, J. Li, L. Lv, Y. Zhao, L. Qu. Vertically aligned graphene sheets membrane for highly efficient solar thermal generation of clean water. ACS Nano **11**(5), 5087-5093 (2017). <https://doi.org/10.1021/acsnano.7b01965>
23. F. Meng, Y. Zhang, S. Zhang, B. Ju, B. Tang. Polysulfide nanoparticles-reduced graphene oxide composite aerogel for efficient solar-driven water purification. Green Energy Environ. **8**(1), 267-274 (2023). <https://doi.org/10.1016/j.gee.2021.04.004>
